# Graph Theoretical Analysis of Functional Brain Networks: Test-Retest Evaluation on Short- and Long-Term Resting-State Functional MRI Data

**DOI:** 10.1371/journal.pone.0021976

**Published:** 2011-07-19

**Authors:** Jin-Hui Wang, Xi-Nian Zuo, Suril Gohel, Michael P. Milham, Bharat B. Biswal, Yong He

**Affiliations:** 1 State Key Laboratory of Cognitive Neuroscience and Learning, Beijing Normal University, Beijing, China; 2 Laboratory for Functional Connectome and Development, Key Laboratory of Behavioral Science, Institute of Psychology, Chinese Academy of Sciences, Beijing, China; 3 Phyllis Green and Randolph Cōwen Institute for Pediatric Neuroscience, New York University Langone Medical Center, New York, New York, United States of America; 4 Department of Radiology, University of Medicine and Dentistry of New Jersey, Newark, New Jersey, United States of America; Newcastle University, United Kingdom

## Abstract

Graph-based computational network analysis has proven a powerful tool to quantitatively characterize functional architectures of the brain. However, the test-retest (TRT) reliability of graph metrics of functional networks has not been systematically examined. Here, we investigated TRT reliability of topological metrics of functional brain networks derived from resting-state functional magnetic resonance imaging data. Specifically, we evaluated both short-term (<1 hour apart) and long-term (>5 months apart) TRT reliability for 12 global and 6 local nodal network metrics. We found that reliability of global network metrics was overall low, threshold-sensitive and dependent on several factors of scanning time interval (TI, long-term>short-term), network membership (NM, networks excluding negative correlations>networks including negative correlations) and network type (NT, binarized networks>weighted networks). The dependence was modulated by another factor of node definition (ND) strategy. The local nodal reliability exhibited large variability across nodal metrics and a spatially heterogeneous distribution. Nodal degree was the most reliable metric and varied the least across the factors above. Hub regions in association and limbic/paralimbic cortices showed moderate TRT reliability. Importantly, nodal reliability was robust to above-mentioned four factors. Simulation analysis revealed that global network metrics were extremely sensitive (but varying degrees) to noise in functional connectivity and weighted networks generated numerically more reliable results in compared with binarized networks. For nodal network metrics, they showed high resistance to noise in functional connectivity and no NT related differences were found in the resistance. These findings provide important implications on how to choose reliable analytical schemes and network metrics of interest.

## Introduction

The human brain is a highly complex system represented as a structurally interconnected network by a dense of cortico-cortical axonal pathways (i.e., structural connectome, [1]) and a functionally synchronized network by external or intrinsic coherent neural activity (i.e., functional connectome, [2]). Mapping the brain connectome and highlighting the underlying organizational principles are fundamental for our understanding of the brain architecture. Recent studies have manifested that human brain connectome networks can be constructed using neuroimaging (e.g., functional MRI (fMRI) and diffusion tensor imaging (DTI)) or electrophysiological (e.g., electroencephalography (EEG) and magnetoencephalography (MEG)) data and further investigated by graph theoretical approaches. These brain networks have consistently demonstrated many non-trivial topological properties, such as small-worldness, modularity and highly connected hubs (for reviews, see [3], [4], [5], [6], [7]), and exhibited distinct alterations associated with different neurocognitive disorders (for reviews, see [8], [9]).

While graph theoretical approaches provide valuable insights into normal brain architecture and pathological mechanism for brain disorders, the test-retest reliability has not been systematically investigated. Reliable measures are fundamental to infer trustworthy conclusions and to serve as potential clinical biomarkers. In response to the demand, several groups examined the TRT reliability/reproducibility of graph network metrics. In anatomical world, Vaessen et al. [10] assessed the reproducibility of anatomical brain networks derived from DTI data and reported high inter-scan reproducibility of network metrics across sampling schemes (e.g., number of gradient directions and gradient amplitude). Bassett et al. [11] demonstrated high reproducibility and low variability of graph metrics for both DTI and diffusion spectrum imaging data derived networks. As for functional imaging arena, Deuker et al. [12] investigated the TRT reliability of functional brain networks using MEG data and reported high reliability during a working memory task but relatively low under resting condition for network metrics. More recently, Telesford et al. [13] constructed functional brain networks using baseline fMRI data during an executive task and demonstrated excellent reproducibility for both small-world properties and network efficiency metrics. Despite these progresses, however, the TRT reliability of network metrics derived from resting-state fMRI (R-fMRI) dataset has not been well documented so far.

R–fMRI is a promising tool to non-invasively map intrinsic functional connectivity patterns of the human brain in vivo [2], [14], [15], [16] and has been extensively used to investigate inherent brain network topological organization (for a review, see [17]). Of note, several previous R-fMRI studies suggest that the strength of interregional functional connectivity is dynamic in time (from seconds to minutes) and frequency domains [18] and can be modulated by the levels of current conscious awareness [19], [20], [21], [22], emotional state [23] and cognitive demand prior to resting-state scanning [24], [25], [26]. Using R-fMRI, Shehzad et al. [27] have demonstrated modest to good TRT reliability for some specific functional connections. However, these states or experiments related alterations in functional connectivity may further interact with the global network topology [28]. To our best knowledge, there are no studies to systematically examine the TRT reliability of network topological metrics derived from R-fMRI data. Accordingly, systematic and direct work is clearly warranted.

In the current study, we implemented a comprehensive estimation of TRT reliability for both global network properties and regional nodal characteristics of intrinsic functional brain networks constructed using a public TRT R-fMRI dataset (http://www.nitrc.org/projects/trt). This dataset allows us to examine both short-term (<1 hour apart) and long-term (>5 months apart) network reliability. Moreover, given numerous discrepancies in the analytical strategies of existing brain network studies (e.g., how to define network nodes or how to deal with negative correlations/connections), we further evaluated the effects of three factors on network reliability. They are: (1) network node definition (ND, i.e, structural regions of interest (ROIs) based node definition or functional ROIs based node definition); (2) network membership (NM, i.e., inclusion or exclusion negative correlations); and (3) network type (NT, i.e., binarized or weighted networks). Table 1 lists those acronyms specific to the current study.

**Table 1:** Brief descriptions of several specific acronyms used in the present study.

Abbreviation	Full name	Explanation
TI	Scanning time interval	Short-term: <1 hour apart Long-term: >5 months apart
NM	Network membership	Network (+): only positive correlations
		Network (+/-): both positive and negative correlations
NT	Network type	Binarized: binarized network
		Weighted: weighted network
ND	Node definition	S-: Structural ROIs
		F-: Functional ROIs
S-AAL	Structural Anatomical Automatic Labeling atlas	This atlas includes 90 regions
S-HOA	Structural Harvard-Oxford atlas	This atlas includes 112 regions
F-DOS	Functional ROIs from ref (40)	This set of ROIs includes 160 regions

## Methods

### Subjects

We used a TRT R-fMRI dataset of 25 participants (mean age 30.7 ± 8.8, 9 males) that is publicly available at NITRC (http://www.nitrc.org/projects/trt). The dataset has been used to examine TRT reliability of seed-based resting-state functional connectivity (RSFC) [27], independent component analysis and dual regression [29], amplitude of low-frequency fluctuations [30] and functional homotopy [31].

### Data acquisition

Three resting-state scans were obtained for each participant using a Siemens Allegra 3.0-Tesla scanner. Each scan consisted of 197 contiguous EPI functional volumes (time repetition (TR) = 2000 ms; time echo (TE) = 25 ms; flip angle (FA) = 90°, number of slices = 39, matrix = 64×64; field of view (FOV) = 192 mm; acquisition voxel size = 3×3×3 mm^3^). Scans 2 and 3 were conducted in a single-scan session, 45 minutes apart, and were 5–16 months (mean 11±4) after scan 1. All individuals were asked to relax and remain still with eyes open during the scan. Additionally, a high-resolution T1-weighted magnetization prepared gradient echo sequence was also obtained (MPRAGE, TR = 2500 ms; TE = 4.35 ms; inversion time = 900 ms; FA = 8°; number of slices = 176; FOV = 256 mm).

### Data preprocessing

Data preprocessing was performed using SPM5 package (http://www.fil.ion.ucl.ac.uk/spm). First, all images were corrected for intra-volume acquisition time offsets between slices using the Sinc interpolation and inter-volume geometrical displacement due to head movement using six-parameter (rigid body) transformation. Then all functional images were normalized into the Montreal Neurological Institute space using an optimum 12-parameter affine transformation and nonlinear deformations, and then resampled to 3-mm isotropic voxels. Finally, the normalized images were further temporally band-pass filtered (0.01–0.1 Hz) to reduce the effects of low-frequency drift and high-frequency physiological noise. Notably, for the extraction of mean nodal time courses of functional defined ROIs, spatial smoothing with 6-mm full width at half maximum (FWHM) Gaussian kernel was performed before band-pass filtering (see below for node definition).

### Functional connectivity matrix and network construction

#### Node definition (ND)

A network (i.e., graph) is comprised of nodes and edges connecting nodes. In the current study, nodes represent ROIs and edges represent RSFC between ROIs. Given the accumulating evidence of effects of node definition on network topology [32], [33], [34], [35], [36], two strategies of defining ROIs (i.e., anatomical and functional ROIs) were employed to provide a comprehensive assessment of TRT reliability of brain networks across different node definitions. Specifically, to obtain structurally defined ROIs, a prior Anatomical Automatic Labeling atlas (AAL) [37] and Harvard-Oxford atlas (HOA) [38], [39] were separately used to divide the whole brain into different number of regions. These two structural atlases parcellated the whole brain into 45 and 56 regions in each hemisphere and were termed as S-AAL and S-HOA, respectively. To obtain functionally defined ROIs, 160 spheres (radius = 5 mm) were generated around the peak coordinates previously identified form meta-analytic studies of multiple brain functions [40], [41] and were termed as F-DOS. These ROIs are comprised of discrete spherical ROIs and not completely cover the cerebral cortex and cerebellum (Fig. S1). All the ROIs are associated with five different kinds of functions of error-processing, default-mode, memory, language and sensorimotor. There were no any overlaps between ROIs. Names of the three sets of ROIs and their corresponding abbreviations are listed in Table S1, S2 and S3.

#### Edge definition

To measure inter-ROI RSFC, for each of the three sets of ROIs, a mean time series for each ROI was calculated by averaging the time series of all voxels within that ROI. Several potential nuisance signals associated with physiological processes were further removed. Specifically, we regressed out estimated head-motion profiles and global signal from each ROI's mean time series [32], [42]. The residuals were then used to estimate inter-ROI RSFC that were quantified by Pearson correlation coefficient. For each subject at each scan, three correlation matrices (corresponding to three sets of ROIs) consisting of Pearson correlation coefficients between each pair of ROIs were therefore generated.

#### Network type (NT)

Individual correlation matrices 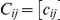 derived above was converted into both a binarized network
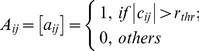
(1) and a weighted network
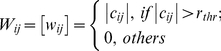
(2) where 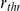
is a pre-defined correlation threshold. To determine 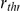, sparsity measure, 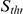 (defined as the ratio of the number of actual edges divided by the maximum possible number of edges in a network) was applied to each correlation matrix. Using the sparsity threshold, a subject-specific 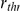 was determined to threshold each correlation matrix such that the resulting networks have the same sparsity level (i.e., the same number of edges) across subjects and scans. Currently, there is no a definitive way to accurately determine threshold and previous studies construct brain networks either under a single threshold (e.g., [43], [44]) or over a continuous threshold range (e.g., [45], [46]) in terms of specific constraint conditions. Here, brain networks were constructed over the full range of sparsity, i.e., 0<
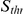
<1 for the whole correlation matrices of both positive and negative correlations and 0<
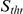
<min [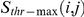] for only positive correlation matrices. 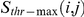 is a data-specific maximum of 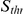 for *i*th subject at *j*th scans (note that scan2 and scan3 were used to determine the threshold range for short-term reliability estimation and scan1 and the average of scan2 and scan3 were used for long-term reliability estimation). Characterization of network topology over continuous sparsity levels allows us to trace the trajectory of TRT reliability of network properties over varying network structures and to identify specific threshold range of high reliability.

#### Network membership (NM)

Given the disagreements in treating negative correlations in R-fMRI network studies (e.g., [46], [47]), the thresholding procedure was performed on both the whole correlation matrices consisting of positive and negative connections and positive correlation matrices consisting of only positive connections (i.e., negative correlations were set to 0).

### Network metrics

We explored two sets of network topological attributes: 1) regional nodal characteristics: degree 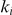, efficiency 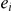, betweenness 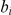, cluster coefficient 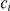, participant coefficient 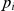, and normalized participant coefficient 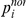; 2) global network metrics: small-world parameters (clustering coefficient 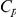, characteristic path length 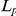, normalized clustering coefficient 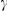, normalized characteristic path length 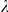 and small-worldness 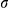), network efficiency (local efficiency 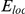 and global efficiency 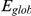), assortativity 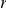, hierarchy 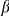, synchronization 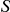, modularity 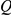 and the number of modules 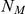. All the computations of network metrics were performed using in-house custom MATLAB codes termed as GRETNA. Text S1 and Table 2 give detailed descriptions for above metrics.

**Table 2:** Brief descriptions of complex network metrics examined in the present study.

Parameter	Character	Descriptions
Regional nodal parameters		
1Degree	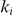	The number of connections linked directly to a node
1Efficiency	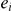	How efficient an index node communicates with the other nodes
2Betweenness	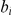	The influences of an index node over information flow between other nodes
1Clustering coefficient	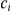	The extent of interconnectivity among the neighbors of an index node
2Participant coefficient	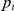	The ability of an index node in keeping the communication between its own module and the other modules
2Normalized 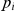	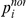	The normalized 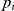 after correcting for the effects of number of modules
Global network parameters		
1Clustering coefficient	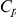	The extent of local clustering or cliquishness of a network
1Characteristic path length	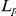	The extent of overall routing efficiency of a network
2Gamma	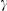	The deviation of 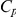 of a network from those of surrogate random networks
2Lambda	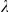	The deviation of 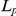 of a network from those of surrogate random networks
2Sigma	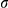	The small-worldness indicating the extent of a network between randomness and order
1Local efficiency	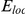	How efficient of information propagation over a node's direct neighbors
1Global efficiency	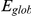	How efficient of information propagation through the whole network
2Assortativity	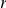	The tendency of nodes to link those nodes with similar number of edges
2Hierarchy	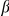	How likely it is that all nodes oscillate with the same wave pattern
1Synchronization	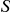	How likely that all nodes fluctuate in the same wave pattern
2Modularity	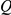	The extent that nodes can be divided several subsets with dense connections within them but sparse between them

Note that the formulas listed here are only for binarized networks. For details for weighted networks, see Text S1.

1 First-order network metrics which are dependent on only one graph property.

2 Second-order network metrics which are dependent on more than one property or are defined as rations of one property.

### Test-retest reliability

To investigate the TRT reliability of all graph metrics mentioned above, we used a common index of intraclass correlation [48]. For each global and nodal network measure derived under each combination of the three factors mentioned above, individual values were first merged into two 25×2 matrices (rows corresponding to subjects and column corresponding to scans), with one representing short-term intra-session across scans 2 and 3 and the other long-term inter-session between scan 1 and the average of scans 2 and 3. Additional long-term reliability estimation using scan 1 and scan 3 alone outputted similar results (Table S4). Therefore, long-term reliability results were reported based on scan 1 and the average of scan 2 and scan 3. Of note, the average was done on individual functional connectivity matrices rather than graph metrics between scan 2 and scan 3, followed by graph metric calculation. Using a one-way ANOVA on each of the two matrices, with random subject effects, we split the total sum of the squares into between-subject (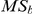) and within-subject (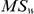, i.e., residual error) sum of squares. Finally, ICC values were calculated according to the following equation where k is the number of repeated observations per subject [48]:
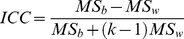
(3)Of note, the ICC derived from (3) has a relationship with the F-value derived from the one-way ANOVA as follows [49]:
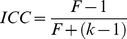
(4)ICC is close to 1 for reliable measures that show low within-subject variance relative to between-subject variance and 0 (negative) otherwise. In the current study, reliability was recorded in terms of the criteria of [50], [51], with an ICC value from 0 to 0.25 indicating poor; 0.25 to 0.4 indicating low; 0.4 to 0.6 indicating fair; 0.6 to 0.75 indicating good and 0.75 to 1.0 indicating excellent reliability. Since the network construction was done over a continuous range of sparsity threshold, ICC is a function of the threshold. To provide a threshold-independent reliability assessment, we also calculated the area under curve (AUC, i.e., the integral) for each network metric [52] that was used to compute a single ICC scalar for each network measure. Finally, we compared the consistency between ICC-based reliability and Pearson correlation coefficient-based similarity analysis (across subjects) for network metrics between scans, which was restricted to S-AAL-based networks.

### Simulation analysis

To investigate the effects of numerical changes in RSFC on network metric reliability, we performed simulation analyses as follows: individual functional connectivity matrices based on S-AAL were calculated using dataset of scan1 and their corresponding network metrics (both global and nodal metrics) were used as reference values. Then, for each correlation matrix, different levels of independent Gaussian noise were added and all network metrics were recomputed. The added Gaussian noise were zero mean and the variances varied across six equally spaced levels corresponding to 8.3%, 16.7%, 25.0%, 33.3%, 41.7% and 50.0% of actual functional connectivity variances for each subject. This procedure assures the same proportion of noise added to each correlation matrix. Of note, the procedure of noise addition was performed 5 times. Therefore, 25 (subjects) 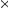 6 (noise levels) 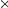 5 (random times) = 750 functional connectivity matrices were simulated in total. Finally, the TRT reliability of each metric was calculated between the reference values and those obtained from simulated functional networks under each level of noise and then averaged across 5 rounds of noise addition.

## Results

### TRT reliability of RSFC: S-AAL

#### Consistency of overall RSFC patterns

**Figure 1:**
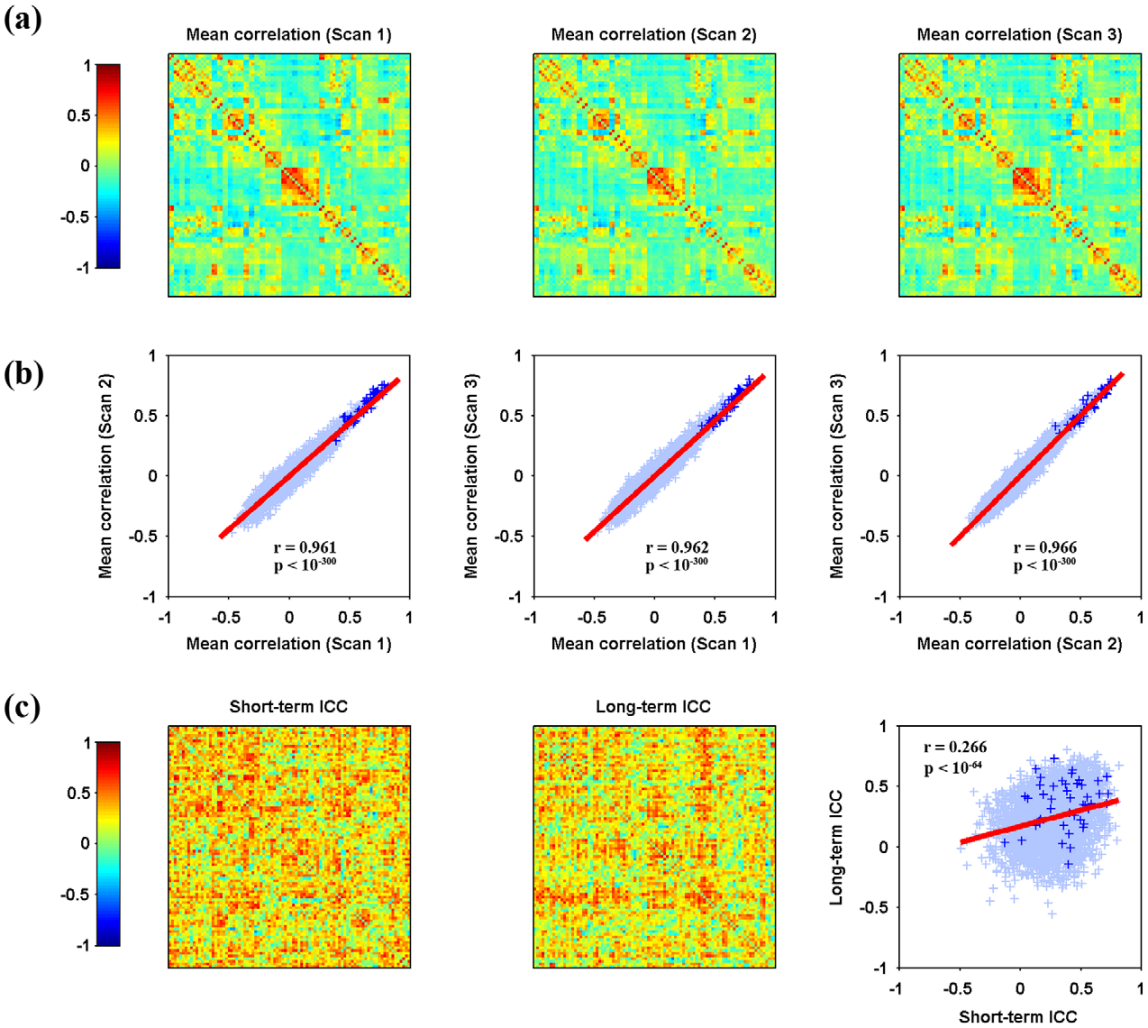
Spatial similarity and TRT reliability patterns of S-AAL-based RSFC. Mean Pearson correlation matrices (a), consistency of overall patterns between mean matrices (b) and TRT reliability of individual connections as well as the relationship between short-term and long-term reliability (c) are illustrated. The mean correlation matrices exhibited high similarity from both visual inspection (a) and quantitative spatial correlation analyses (b). Further TRT reliability analyses revealed a portion of connections exhibiting fair to excellent reliability (c, also see Fig. 2). Moreover, short-term reliability was significantly (p<0.05) correlated with long-term reliability among connections (c). Functional connections linking inter-hemisphere homotopic regions, as highlighted by plus signs (+), showed high connectivity strength and many of them exhibited high reliability. TRT, test-retest; RSFC, resting-state functional connectivity; S-AAL, structural ROIs from Anatomical Automatic Labeling atlas. Of note, the structural ROIs were listed as in Table S1.

The mean RSFC matrices across subjects were calculated (after Fisher's r-to-z transform) for each of the three TRT scans. Initial visual inspection suggested that mean RSFC matrices showed highly similar spatial patterns between different time points (Fig. 1a). Further quantitative spatial correlation analysis (Pearson correlation) confirmed the visual inspection, as revealed by high correlations in the mean correlation values between each pair among the three scans (Fig. 1b, scan1 vs. scan2: r = 0.961, p<10^−300^; scan1 vs. scan3: r = 0.962, p<10^−300^; scan2 vs. scan3: r = 0.966, p<10^−300^).

#### Reliability of RSFC

**Figure 2:**
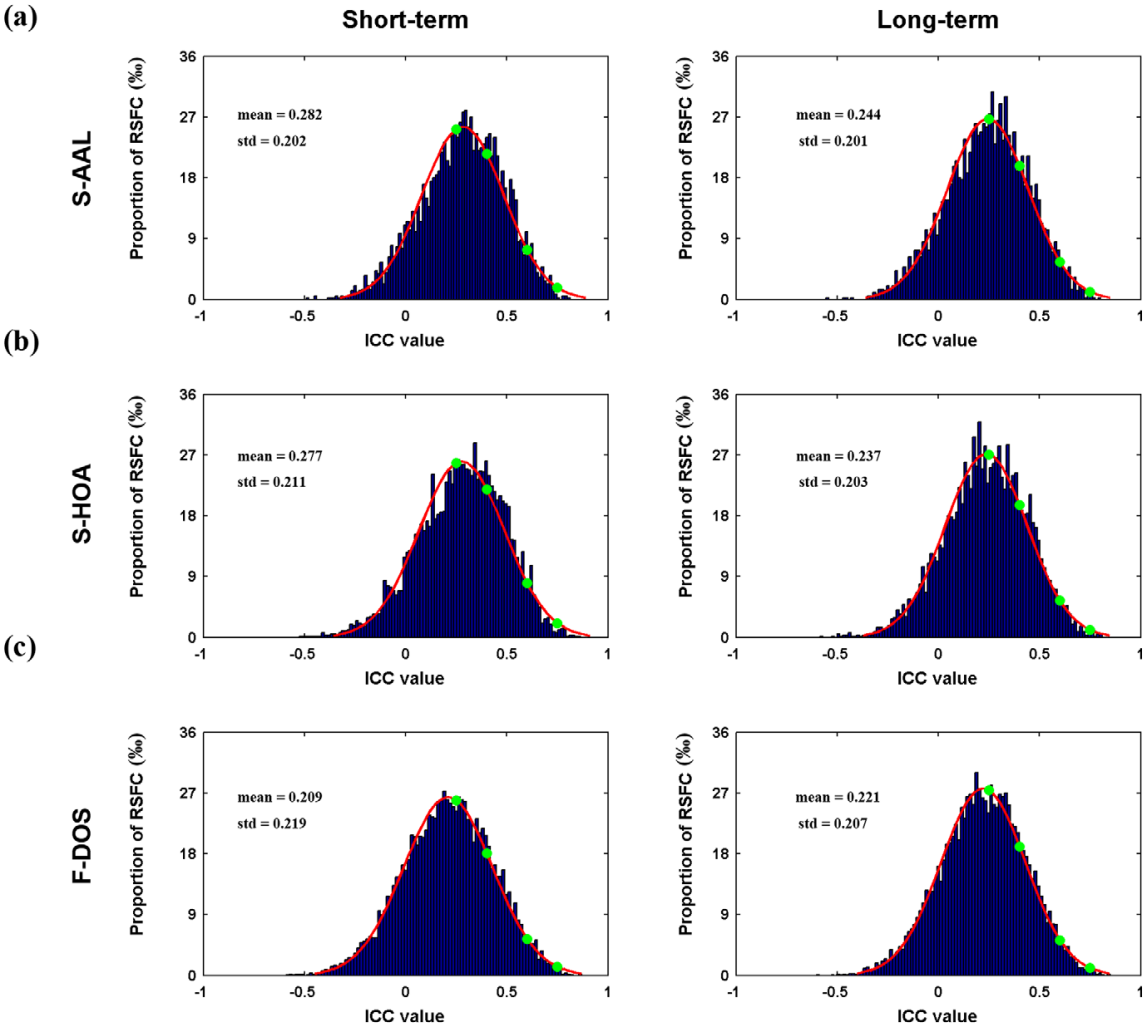
TRT reliability distribution of RSFC. Both short-term and long-term TRT reliability exhibit approximatively normal distribution for all ROI sets. The mean reliability was about 0.28 (short-term) and 0.24 (long-term) for both structural ROIs-based RSFC while relatively low values were observed for functional ROIs-based RSFC. Green dots indicate the critical values used in the present study to grade reliability. RSFC, resting-state functional connectivity; TRT, test-retest.

ICC-based TRT reliability analysis on individual functional connections demonstrated an approximate normal distribution of the ICC values for all 4005 (i.e., 90×89/2) connections with a mean around 0.25 for both short-term and long-term scans (Fig. 2a). In terms of the category used in the present study, 1203 (∼30.0%) functional connections exhibited fair to excellent reliability for short-term scans (fair: 1006, ∼25.1%; good: 191, ∼4.8%; excellent: 6, ∼0.2%) and 914 connections (∼22.8%) for long-term scans (fair: 796, ∼19.9%; good: 114, ∼2.9%; excellent: 4, ∼0.1%). The majority of RSFC (up to 70% for both short- and long-term scans) showed low or poor reliability. Additionally, a significantly positive correlation (Pearson correlation, r = 0.266, p<10^−64^) was found in the ICC values across connections between short-term and long-term scans (Fig. 1c).

#### Relationship between connectivity and reliability

**Figure 3:**
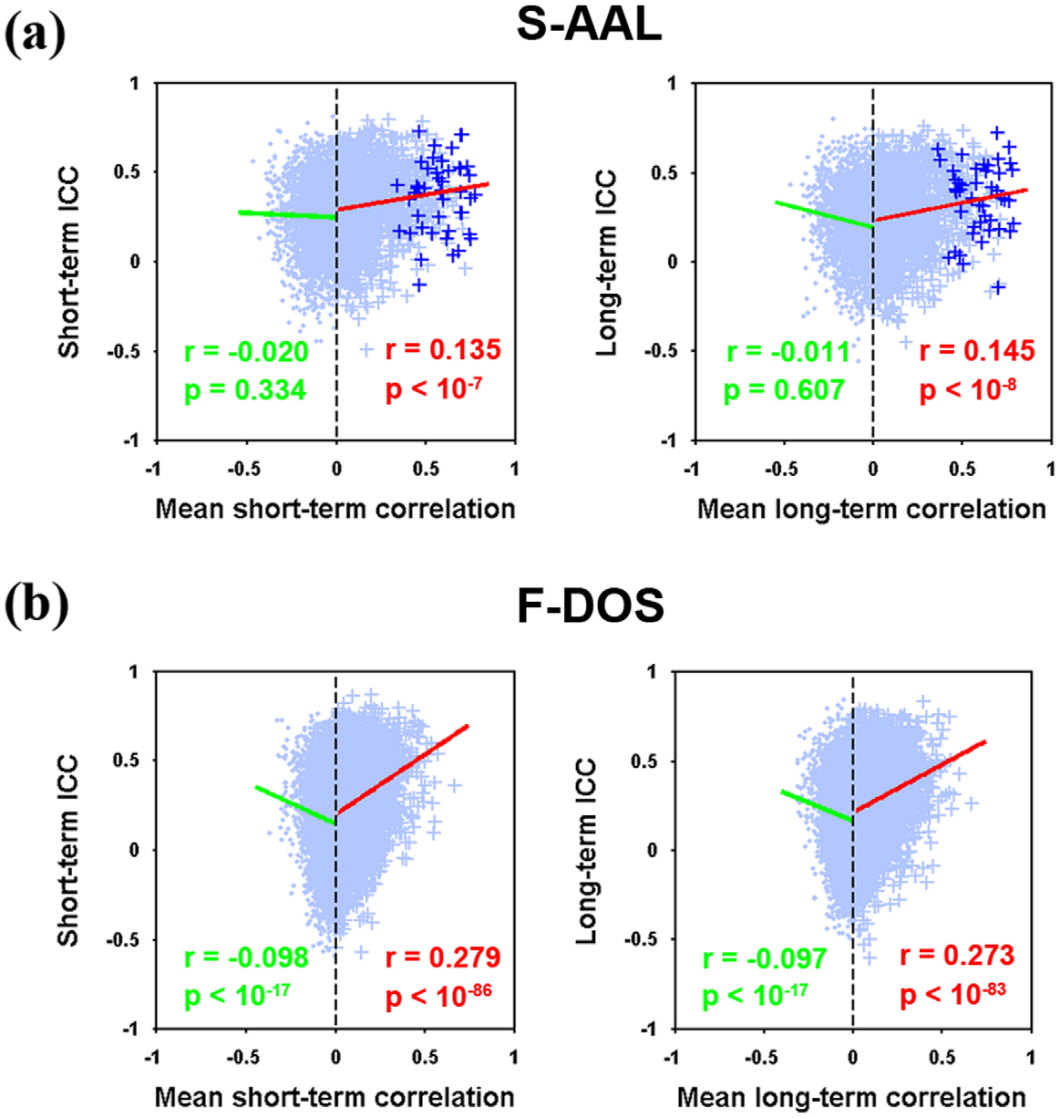
Relationship between RSFC and TRT reliability. Scatter plots of mean connectivity strength against corresponding ICC values are depicted to show the relationship for both S-AAL (a) and F-DOS (b) based correlation matrices. The trend lines were obtained by linear least-square fit. Significant (p<0.05) positive correlations were found between positive RSFC and their corresponding ICC values for both ROIs sets and for both short-term and long-term scanning. In addition, significant negative correlations were also found for negative RSFC with their corresponding ICC values but only for F-DOS-based correlation matrices. These findings suggest higher reliability for stronger RSFC. Functional connections linking inter-hemisphere homotopic regions are highlighted by plus signs (+) for S-AAL but not for F-DOS because of the absence of direct correspondence. RSFC, resting-state functional connectivity; TRT, test-retest; S-AAL, structural ROIs from Anatomical Automatic Labeling atlas; F-DOS, functional ROIs from Dosenbach et al. (2006, 2010).

To explore the relationship between connectivity strength and reliability, linearly fitted lines were obtained separately for positive connections and negative connections with their corresponding ICC values. We found significantly positive correlations (Pearson correlation) between positive connections and their ICC values for both short-term (r = 0.135, p<10^−7^) and long-term (r = 0.145, p<10^−8^) scans (Fig. 3a). No significant correlations were found between negative correlations and their ICC values (p>0.3 for both short-term and long-term scans) (Fig. 3a). These findings indicate that reliability of functional connectivity was partly determined by their strength, whereas functional connectivity strength had limited predictive ability to their reliability since the small amount of variance in the functional connectivity reliability explained by their strength (R^2^<3%).

### TRT reliability of RSFC: S-HOA

#### Consistency of overall RSFC patterns

The mean S-HOA-based RSFC matrices across subjects also showed highly similar spatial patterns revealed by visual inspection (Fig. S2a) and quantitative spatial correlation analyses (Fig. S2b, r>0.95 between any two scans).

#### Reliability of RSFC

Similar to S-AAL, approximate normal distributions were also found for the reliability of S-HOA-based RSFC which had comparable mean (∼ 0.25) (Fig. 2b). Also consistent with S-AAL, although there were quite a few connections showing fair to good to excellent reliability (1874, ∼30.1% for short-term and 1356, ∼21.8% for long-term scans), most connections were poorly reliable (4342, ∼69.9% for short-term and 4860, ∼78.2% for long-term scans). Finally, short-term reliability was found to positively correlate with long-term reliability across connections (Fig. S2c, Pearson correlation, r = 0.307, p<10^−135^).

#### Relationship between connectivity and reliabiflity

Consistent with S-AAL, positive correlations were found between positive RSFC and their reliability for the S-HOA-based correlations (short-term: r = 0.166, p<10^-17^; long-term: r = 0.148, p<10^−13^), indicating limited determination of functional confnectivity strength on their reliability (R^2^<3%). Additionally, a negative correlation was demonstrated between negative correlations and their reliability for long-term scans (r = −0.033, p = 0.048) (Fig. S3).

### TRT reliability of RSFC: F-DOS

#### Consistency of overall RSFC patterns

**Figure 4:**
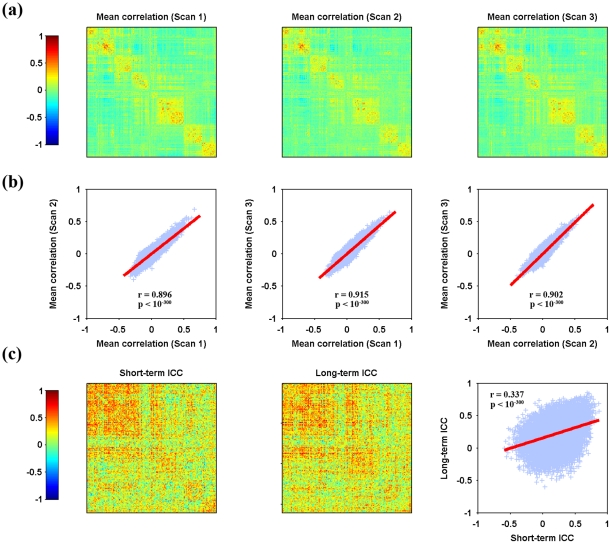
Spatial similarity and TRT reliability patterns of F-DOS-based RSFC. Mean Pearson correlation matrices (a), consistency of overall patterns between mean matrices (b) and TRT reliability of individual connections as well as the relationship between short-term and long-term reliability (c) are illustrated. The mean correlation matrices exhibited high similarity from both visual inspection (a) and quantitative spatial correlation analyses (b). Further TRT reliability analyses revealed many connections exhibiting fair to excellent reliability (c, also see Fig. 2). Moreover, a significant (p<0.05) correlation was found in the ICC matrices between short-term and long-term scans (c). No inter-hemisphere homotopic functional connections were highlighted because of the absence of direct inter-hemisphere correspondence for these ROIs. TRT, test-retest; RSFC, resting-state functional connectivity; F-DOS, functional ROIs from Dosenbach et al. (2006, 2010). Of note, the functional ROIs were listed as in Table S3.

Relative to structural ROIs-based RSFC matrices (both S-AAL and S-HOA), the similarity in the spatial patterns across scans decreased for the mean RSFC matrices derived on the basis of 160 functional ROIs but still remained high (Scan1 vs. Scan2: r = 0.896, p<10^−300^; Scan1 vs. Scan3: r = 0.915, p<10^−300^; Scan2 vs. Scan3: r = 0.902, p<10^−300^) (Fig. 4a and b).

#### Reliability of RSFC

Normal distributions were also found for TRT reliability of functional ROIs-based RSFC, however lower mean ICC values (∼0.20) were obtained in this case in comparison with structural ROIs-based RSFC (∼0.25) (Fig. 2c). Moreover, higher percentage of connections (up to ∼80.0%) showed poor and low reliability for both short-term and long-term scanning procedure, with ∼20.0% showing fair to good to excellent reliability. Of note, those reliable connections were mainly related with ROIs designated as default mode network according to previous study [40]. In addition, short-term reliability was found to positively correlate with long-term reliability across all connections (Fig. 4c, Pearson correlation, r = 0.337, p<10^-300^).

#### Relationship between connectivity and reliability

In the case of functional ROIs based RSFC matrices, functional connectivity strength explained relatively more in comparison with structural ROIs based matrices but still low variance (R^2^<8%) in connectivity reliability (positive correlations and their reliability: r = 0.279, p<10^-86^ for short-term and r = 0.273, p<10^-83^ for long-term scans; negative correlations and their reliability: r = −0.098, p<10^−17^ for short-term and r = −0.097, p<10^−17^ for long-term scans) (Fig. 3b).

### TRT reliability of network metrics: S-AAL

#### Reliability of global network metrics

**Figure 5:**
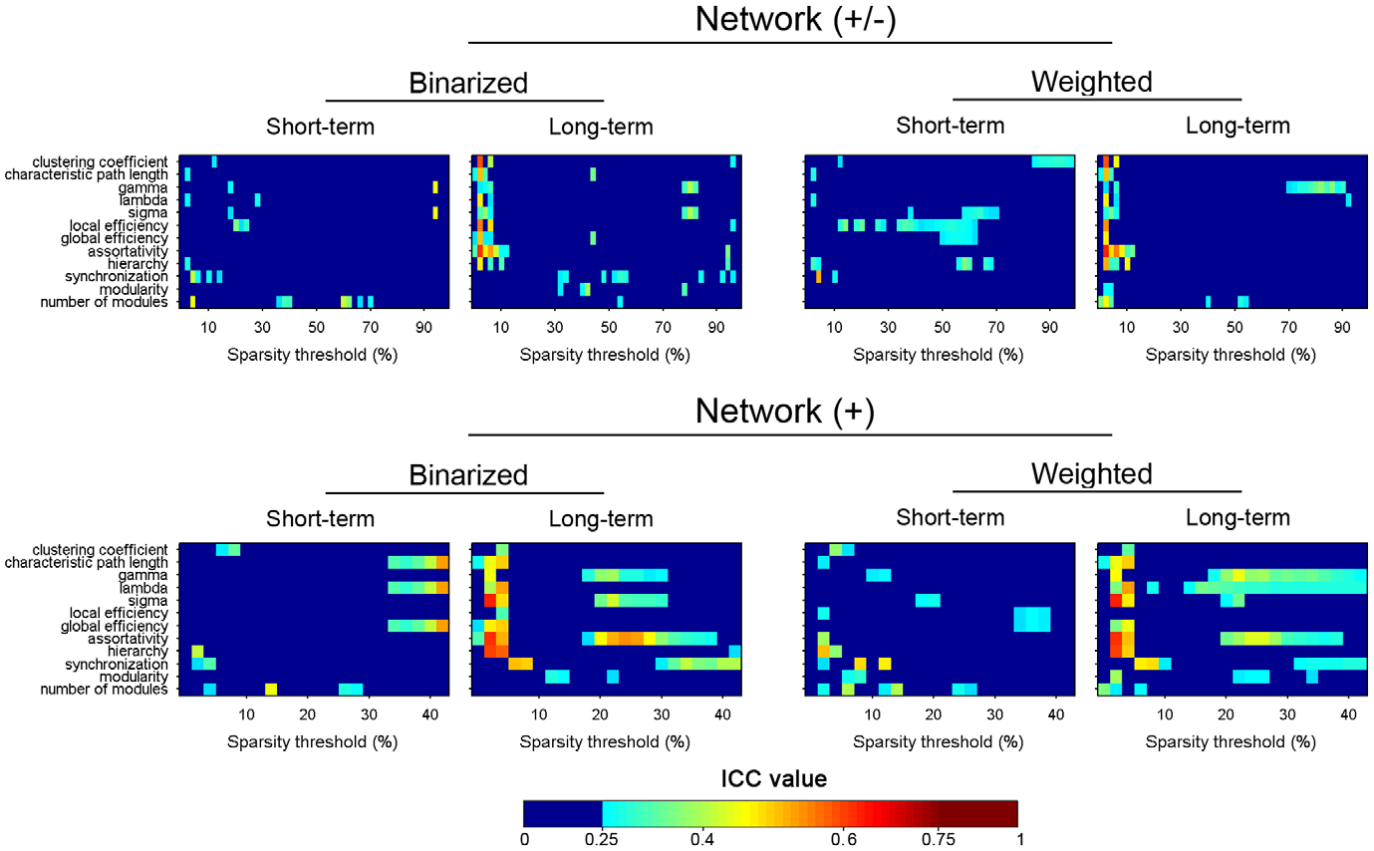
TRT reliability of global network metrics as a function of sparsity threshold for S-AAL-based networks. ICC values less than 0.25 were mapped to a single color of dark blue as well dark red color for ICC values greater than 0.75, respectively. Network (+/-), networks constructed using absolute both positive and negative correlations; Network (+), networks constructed using only positive correlations; Binarized, binarized network anlysis; Weighted, weighted network analysis; TRT, test-retest; S-AAL, structural ROIs from Anatomical Automatic Labeling atlas.

**Figure 6:**
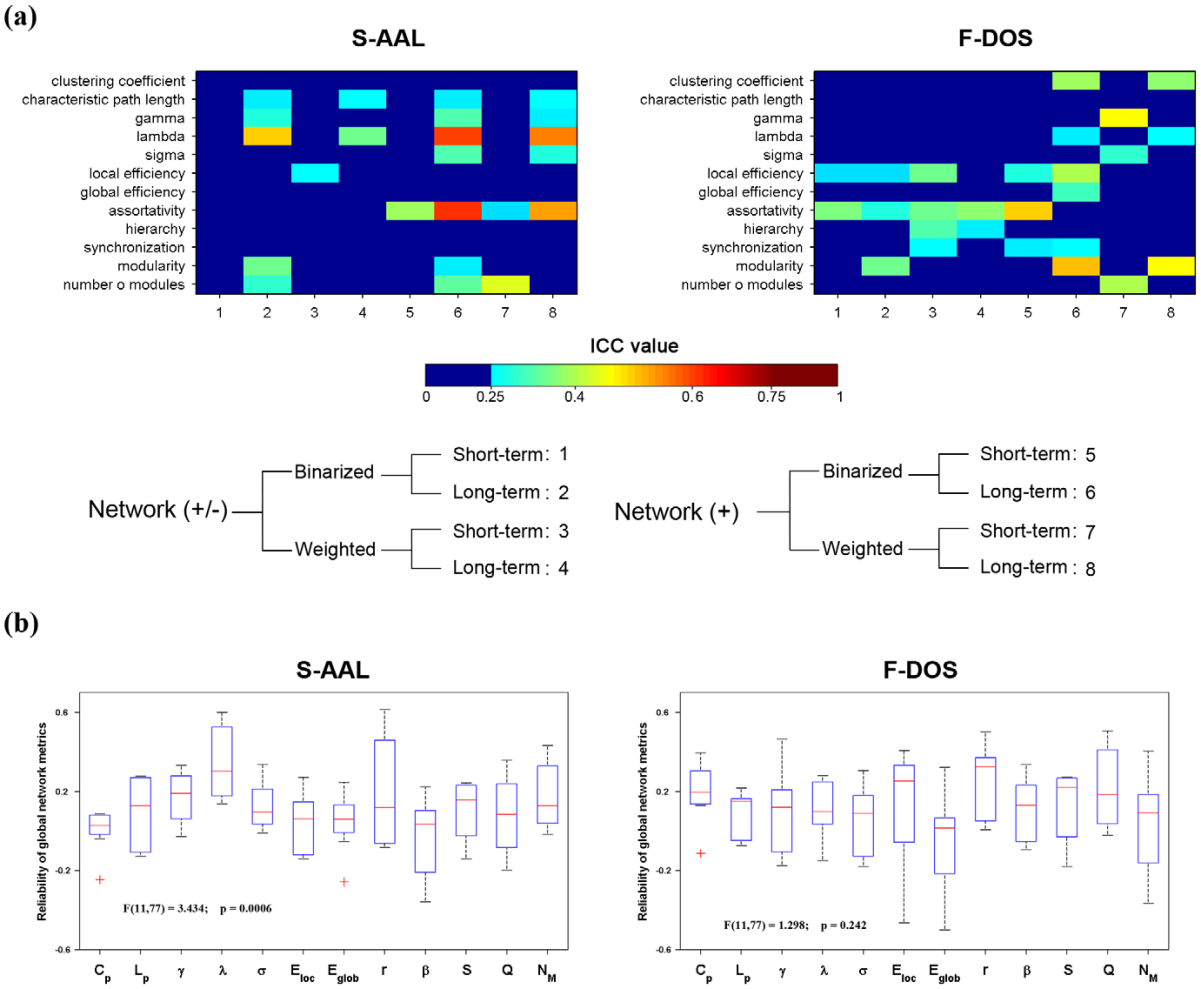
TRT reliability of summarized global network metrics (a) and metric-related differences in reliability (b). Areas under curves (AUCs) of each metrics were used to provide threshold-independent reliability estimation. Different metrics showed variable levels of reliability. Several of them were moderately reliable (e.g., lambda for S-AAL-based networks). Subsequent statistical analysis revealed significant differences in TRT reliability among the 12 global network metrics for S-AAL- but not for F-DOS-based networks. ICC values less than 0.25 were mapped to a single color of dark blue as well dark red color for ICC values greater than 0.75, respectively in (a). Network (+/-), networks constructed using absolute both positive and negative correlations; Network (+), networks constructed using only positive correlations; Binarized, binarized network analysis; Weighted, weighted network analysis; TRT, test-retest; S-AAL, structural ROIs from Anatomical Automatic Labeling atlas; F-DOS, functional ROIs from Dosenbach et al. (2006, 2010).

In the present study, individual networks were constructed at the same sparsity level by applying subject-specific correlation thresholds to individual correlation matrices (see Fig. S4 for the corresponding correlation thresholds under each sparsity level). Sparsity threshold ensures all resultant networks to have comparable topological structures of the same number of edges. Figure 5 shows the TRT reliability of 12 global network metrics over the whole sparisty range. Generally, most global network metrics exhibited poor to low reliability, irrespective of the factors of TI, NT and NM. For example, clustering coefficient 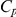 was found to uniformly exhibit poor reliability (ICC<0.25) under all conditions. Nonetheless, we noted that some global metrics (e.g., lambda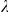 and assortativity 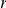) exhibited modest long-term reliability when the networks were sparsely connected (sparsity<10%). Interestingly, we found that global network reliability appeared to depend on the factors of TI and NM but relatively insensitive to NT by qualitatively visual inspection. Specifically, long-term scans seemed to be associated with better reliability in compared with short-term scans and the exclusion of negative correlations enhanced network reliability (Fig. 5). These were reflected in both increased ICC values and the broadened threshold range of high ICC. Finally, a threshold-independent reliability scalar was obtained for each global network metric by using the AUC. Again, several specific global metrics (e.g., lambda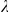) demonstrated moderate long-term reliability under certain analytical schemes (Fig. 6a, left). Of note, we found that assortativity 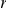
showed moderated both short-term and long-term reliability for networks of positive correlations.

Beyond the descriptive results mentioned above, we further performed statistical analyses to test the differences in reliability among 12 global metrics (one-way repeated-measure ANOVA) and the effects of TI, NM and NT on the reliability of global network metrics (three-factor repeated-measure ANOVA). Those AUC-based ICC values were used for the statistical analyses. The results showed that TRT reliability differed significantly (F(11,77) = 3.434, p = 0.001) among 12 global network metrics with lambda 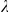 showing the highest reliability (Fig. 6b, left). Furthermore, TI (F(1,11) = 8.176, p = 0.016) and NM (F(1,11) = 4.492, p = 0.058) showed significant or marginally significant main effects on global network reliability, respectively. In addition, a significant interaction was observed between TI and NT (F(1,11) = 5.317 , p = 0.042). NT and other interactions were not significant (p>0.05). Further post-hoc comparisons (paired t-tests) revealed that long-term scans outperformed short-term scans only for binarized networks (t(23) = 5.100, p<10^−4^) but not for weighted networks (t(23) = 1.333, p = 0.196) and excluding negative correlations increased the reliability (t(47) = 3.228, p = 0.002) of global network metrics. See Table 3 for the summary of all statistical results.

**Table 3:** Summary of the main findings in the present study.

	Factors effecting network metric reliability		
	Time interval	Network type	Network membership
S-AAL			
Global metrics	Long>Short (Binarized)	N.S.	Network (+)>Network (+/-)
Nodal metrics	N.S.	N.S.	N.S.
S-HOA			
Global metrics	N.S.	Binarized>Weighted	N.S.
Nodal metrics	N.S.	N.S.	N.S.
F-DOS			
Global metrics^a^	N.S.	N.S.	N.S.
Nodal metrics	N.S.	N.S.	N.S.

S-AAL, structural ROIs from Anatomical Automatic Labeling atlas; S-HOA, structural ROIs from Harvard-Oxford atlas; F-DOS, functional ROIs from ref (40); Network (+/-), networks constructed using absolute both positive and negative correlations; Network (+), networks constructed using only positive correlations; Binarized, binarized network analysis; Weighted, weighted network analysis; N.S., non-significant.

a There were no significant differences in reliability among global network metrics for F-DOS based networks while nodal network metrics for F-DOS and both global and nodal metrics for S-ALL and S-HOA based networks were significantly different.

#### Reliability of local nodal metrics

**Figure 7:**
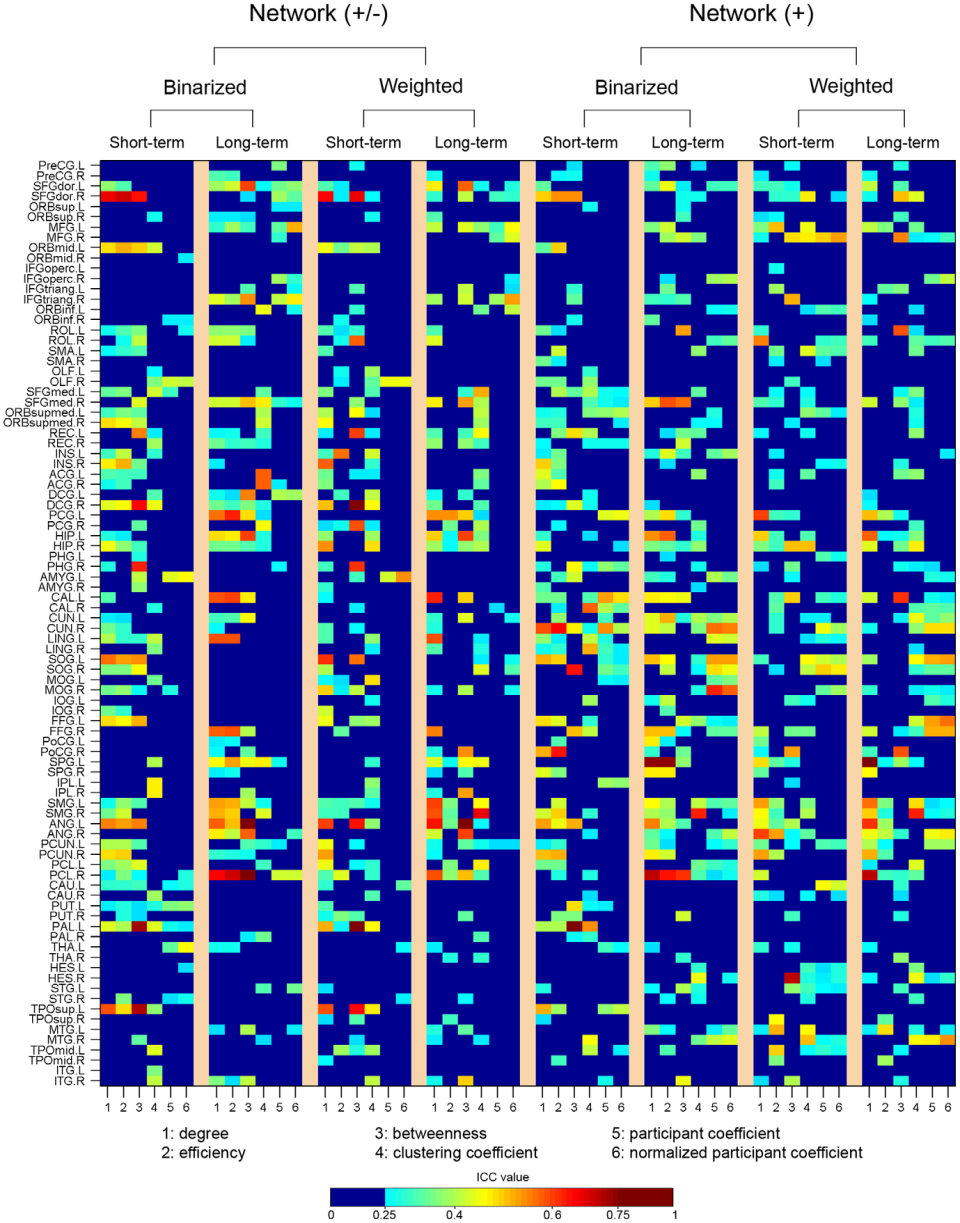
TRT reliability of nodal metrics for S-AAL-based networks. Nodal reliability varied across nodal attributes and spatial locations. The full names of region's abbreviations were listed as in Table S1. ICC values less than 0.25 were mapped to a single color of dark blue as well dark red color for ICC values greater than 0.75, respectively. Network (+/-), networks constructed using absolute both positive and negative correlations; Network (+), networks constructed using only positive correlations; Binarized, binarized network analysis; Weighted, weighted network analysis; TRT, test-retest; S-AAL, structural ROIs from Anatomical Automatic Labeling atlas.

Nodal reliability was estimated based on AUCs. We found that nodal reliability showed: (1) unconspicuous differences associated with factors of TI, NM and NT; (2) different patterns across nodal metrics; and (3) a spatially heterogeneous distribution over the whole brain (Fig. 7).

First, nodal reliability patterns did not show remarkable differences associated with the factors of TI, NM and NT by visual inspection. To test whether or not there exist differences in the TRT reliability associated with these factors, three-factor repeated-measure ANOVA was further performed on the mean ICC values over all nodes. Results revealed that none of these three factors had significant main effects or interactions on the mean nodal reliability (p>0.05) (Table 3).

**Figure 8:**
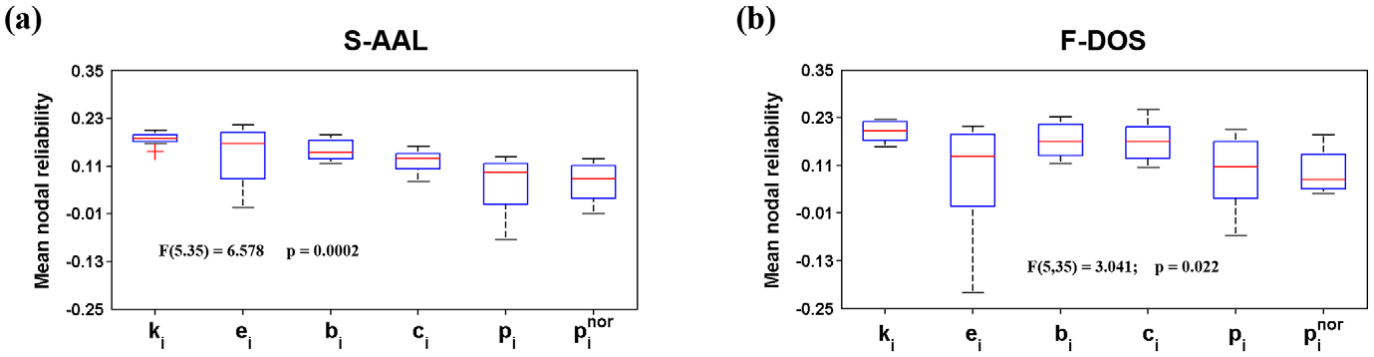
Boxplot of mean nodal TRT reliability for S-AAL- (a) and F-DOS- (b) based networks. Significant differences were found in the mean nodal reliability among the six nodal metrics examined with nodal degree showing the highest ICC values and least variances for both ROIs sets. TRT, test-retest; S-AAL, structural ROIs from Anatomical Automatic Labeling atlas; F-DOS, functional ROIs from Dosenbach et al. (2006, 2010).

Second, nodal reliability exhibited variable patterns across nodal attributes under each combination of the three factors. Further one-factor repeated-measure ANOVA on the mean nodal reliability over regions supported this finding that there was significant (F(5,35) = 6.578, p = 0.0002) differences among the six nodal metrics examined, with the highest ICC values and least variance for nodal degree (Fig. 8a).

**Figure 9:**
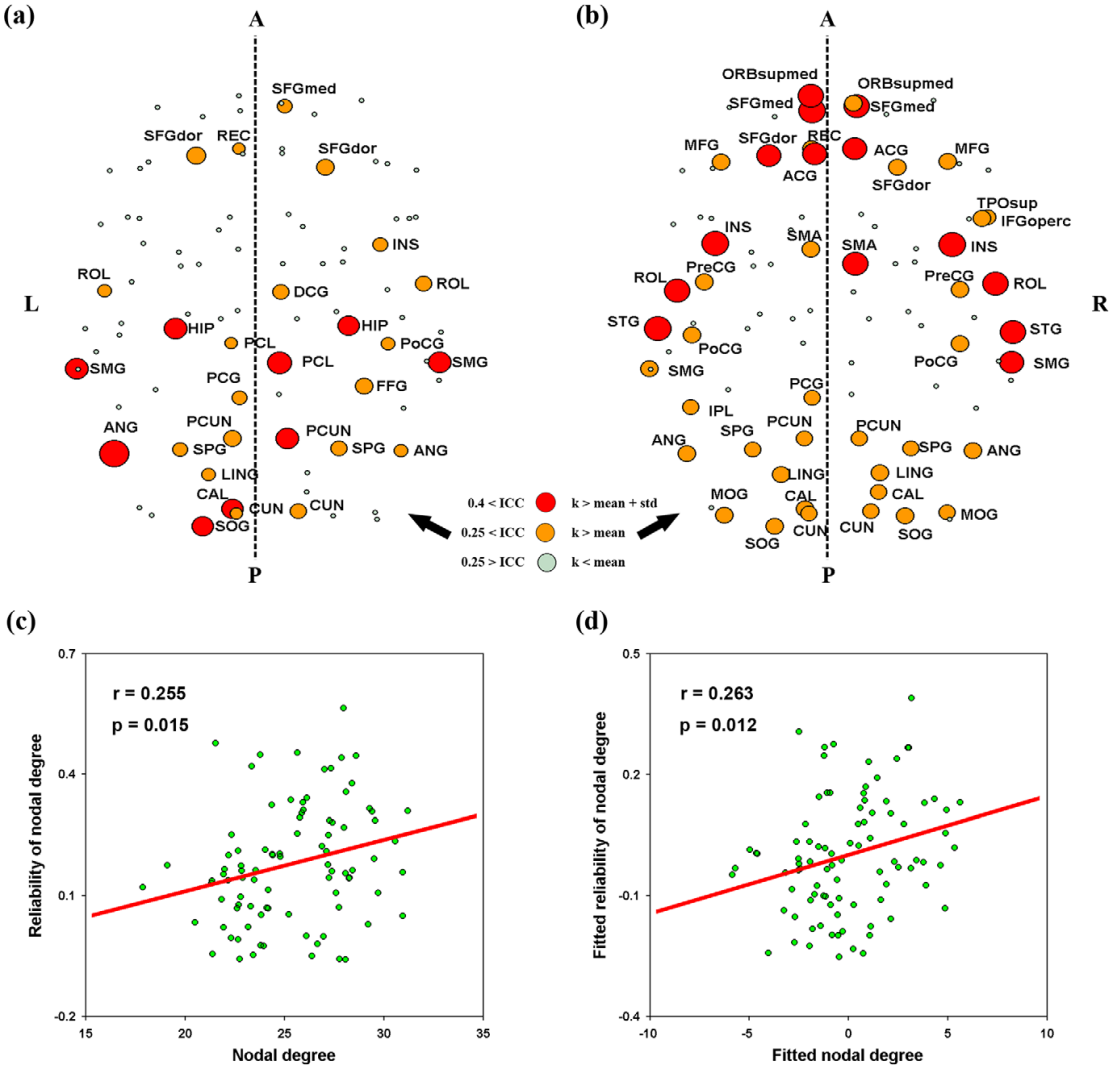
Nodal TRT reliability of degree and its relationship with nodal degree centrality for S-AAL-based networks. (a) Nodal TRT reliability was mapped in anatomical space after average across scanning time interval, network type and network membership because of no effects of these factors on nodal reliability. (b) Nodal degree centrality (AUCs) was also mapped in anatomical space which was averaged across subjects and factors of scanning time interval, network type and network membership. Trend lines were further obtained by linear least-square fit to reveal the relationship between nodal degree centrality and their corresponding reliability after with (d) and without (c) correcting for the effects of regional size. Of note, the full names of region's abbreviations were listed as in Table S1. TRT, test-retest; S-AAL, structural ROIs from Anatomical Automatic Labeling atlas; k, nodal degree; A, anterior; P, posterior; L, left; R, right.

Finally, nodal reliability distributed non-uniformly over the brain, an observation irrespective of nodal metrics and factors of TI, NM and NT. To highlight those reliable regions, we selectively mapped nodal reliability of degree of all regions after averaging over factors of TI, NM and NT (Fig. 9a). This was because nodal degree showed higher reliability and less variance as compared to other nodal metrics and was robust to TI and NM as well as NT. As shown in Figure 9a, some association and limbic/paralimbic cortex regions [53] exhibited fair reliability that were predominately located in bilateral parietal and occipital lobes, such as association cortex regions of the left angular gyrus (ANG), right paracentral lobule (PCL), right precuneus (PCUN), bilateral supramarginal gyrus (SMG), bilateral dorsolateral superior frontal gyrus (SFGdor), right medial superior frontal gyrus (SFGmed) and left superior occipital gyrus (SOG), and limbic/paralimbic regions of the bilateral hippocampus (HIP) and the left posterior cingulate gyrus. In addition, one primary cortex region of the left calcarine fissure (CAL) was also found to be fairly reliable. To test whether or not nodal reliability was related with nodal centrality, we also mapped the mean nodal degree over TI, NM and NT (Fig. 9b) and found visually different patterns between nodal degree and nodal reliability. The most reliable regions located on the posterior while the most connected regions on the anterior portions of the brain. Further quantitative correlation analysis revealed that only tiny variance (R^2^<7%) in nodal reliability could be explained by nodal degree centrality in both cases of with (r = 0.255, p = 0.015, Fig. 9c) and without (r = 0.263, p = 0.012, Fig. 9d) correction for regional nodal size. To test whether there exist a relationship between spatial location and nodal reliability, we compared nodal degree reliability between anterior (y>0) and posterior (y<0) regions. The results revealed that posterior regions were more reliable than anterior regions even if nodal mean functional connectivity differences were corrected (t(87) = 2.801, p = 0.006). In addition, we also found dramatically different patterns across nodal metrics even for those most reliable regions except for the right PCL (Fig. S5a).

#### Consistency between ICC-based reliability analysis and Pearson correlation analysis

**Figure 10:**
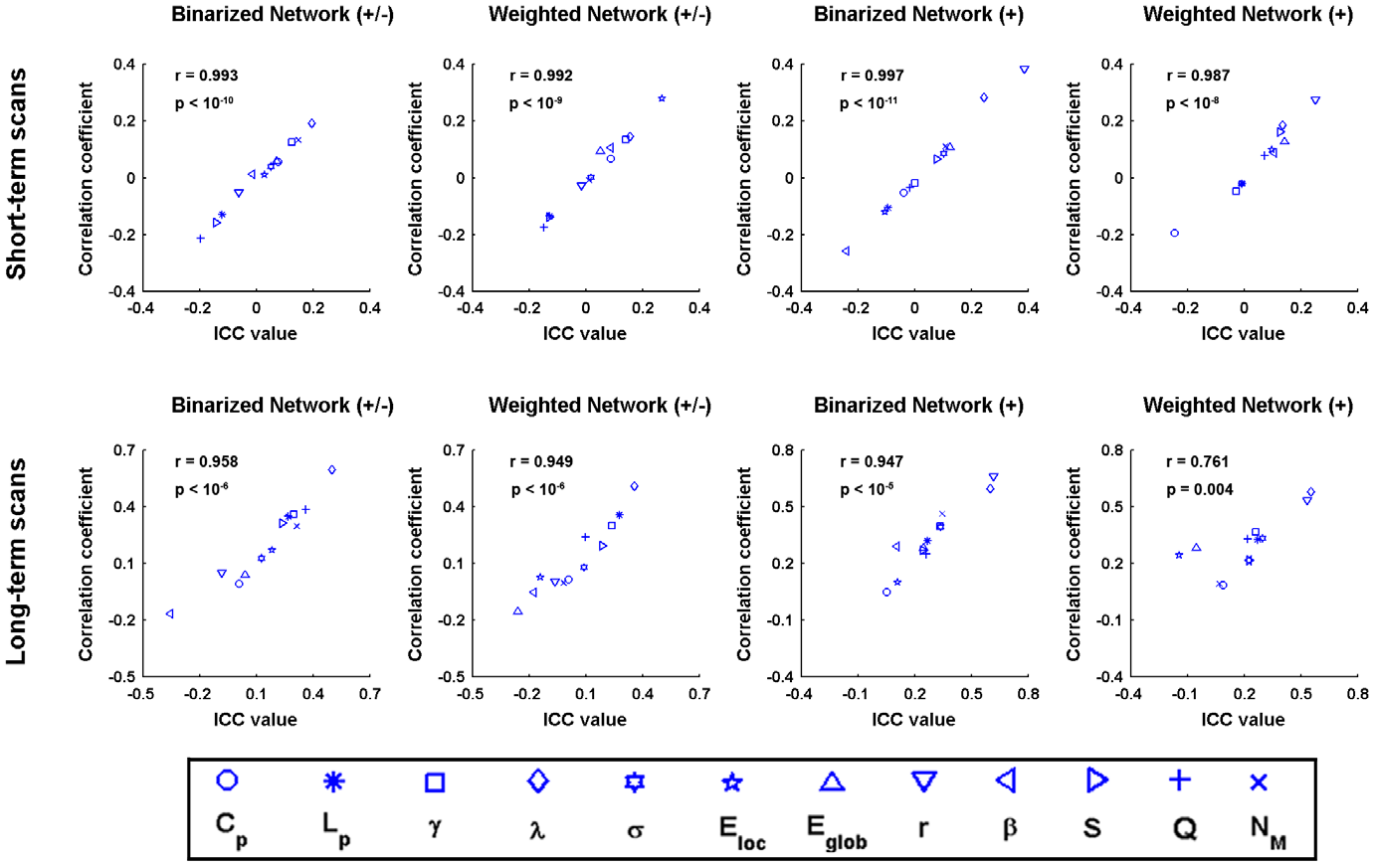
The similarity between inter-scan ICC-based reliability and inter-scan Pearson correlation coefficients for S-AAL-based networks. The reliability and correlation analyses revealed highly consistent results (r>0.9 under most conditions), ruling out the possibility of linear scaling biases of network metrics across test and retest scans that will lead to low TRT reliability.

To test the possibility of linear scaling biases across test and retest scans which may result in low TRT reliability, we calculated the inter-scan Pearson correlation coefficient for each global network metric (AUC) across subjects for both short-term and long-term scans. Further scatter plots between ICC values and Pearson correlation coefficients revealed highly correlated patterns (r>0.9 under most conditions) (Fig. 10), suggesting consistent results revealed by the two measures.

#### Simulation results

**Figure 11:**
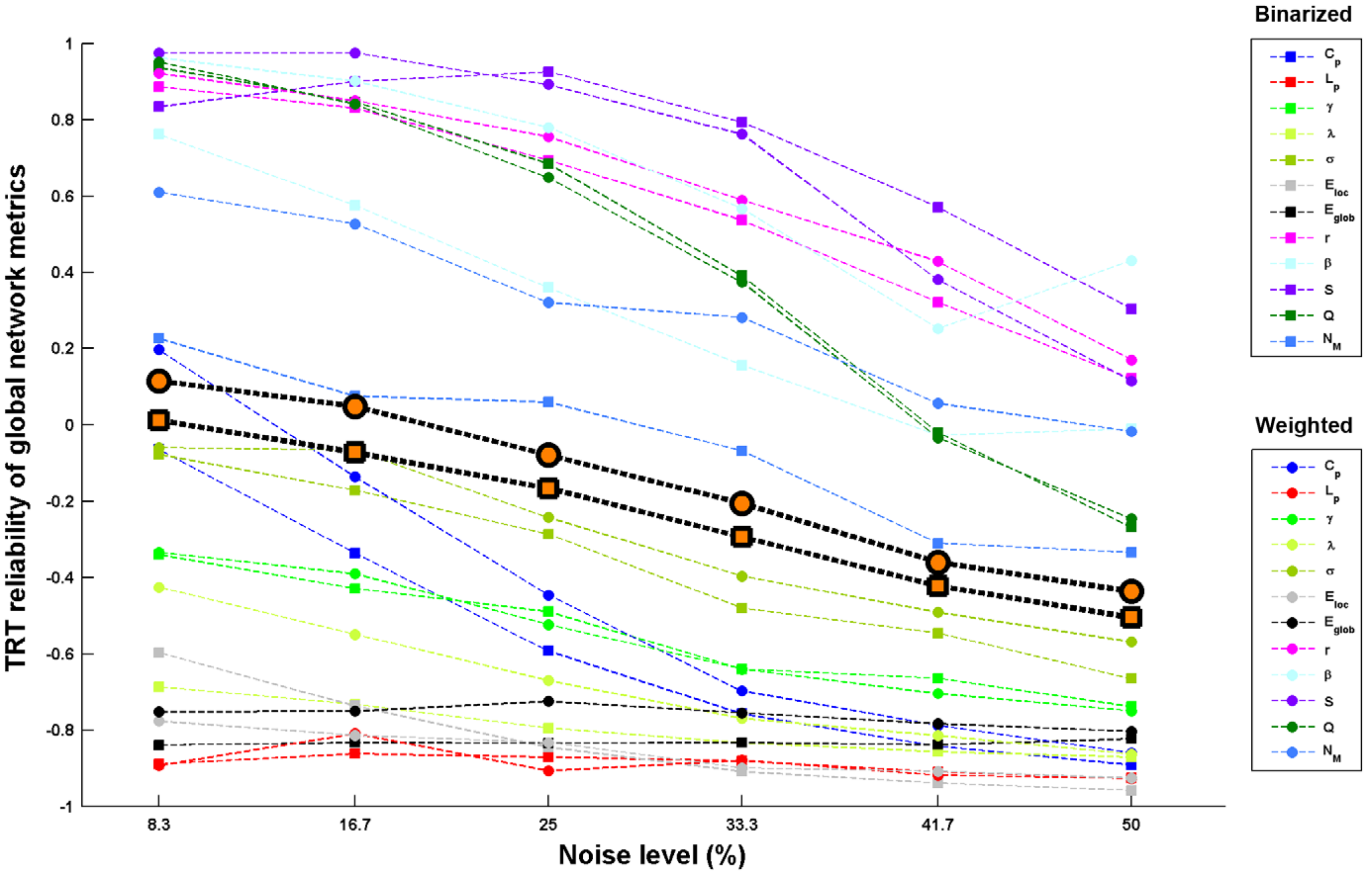
TRT reliability of global network metrics as a function of noise in RSFC for S-AAL-based networks. Global network metrics were sensitive to disturbances of RSFC and weighted network analysis generated numerically more stable results in comparison with binarized network analysis. The highlighted black border marks are the average reliability across metrics for binarized (square) and weighted (circle) network analysis, respectively. Of note, the sensitivity varied dramatically among metrics. Small-world parameters and network efficiency were extremely sensitive to even little noise in functional connectivity while assortativity, hierarchy, synchronization and modularity were relatively resistant to noise. TRT, test-retest; RSFC, resting-state functional connectivity.

**Figure 12:**
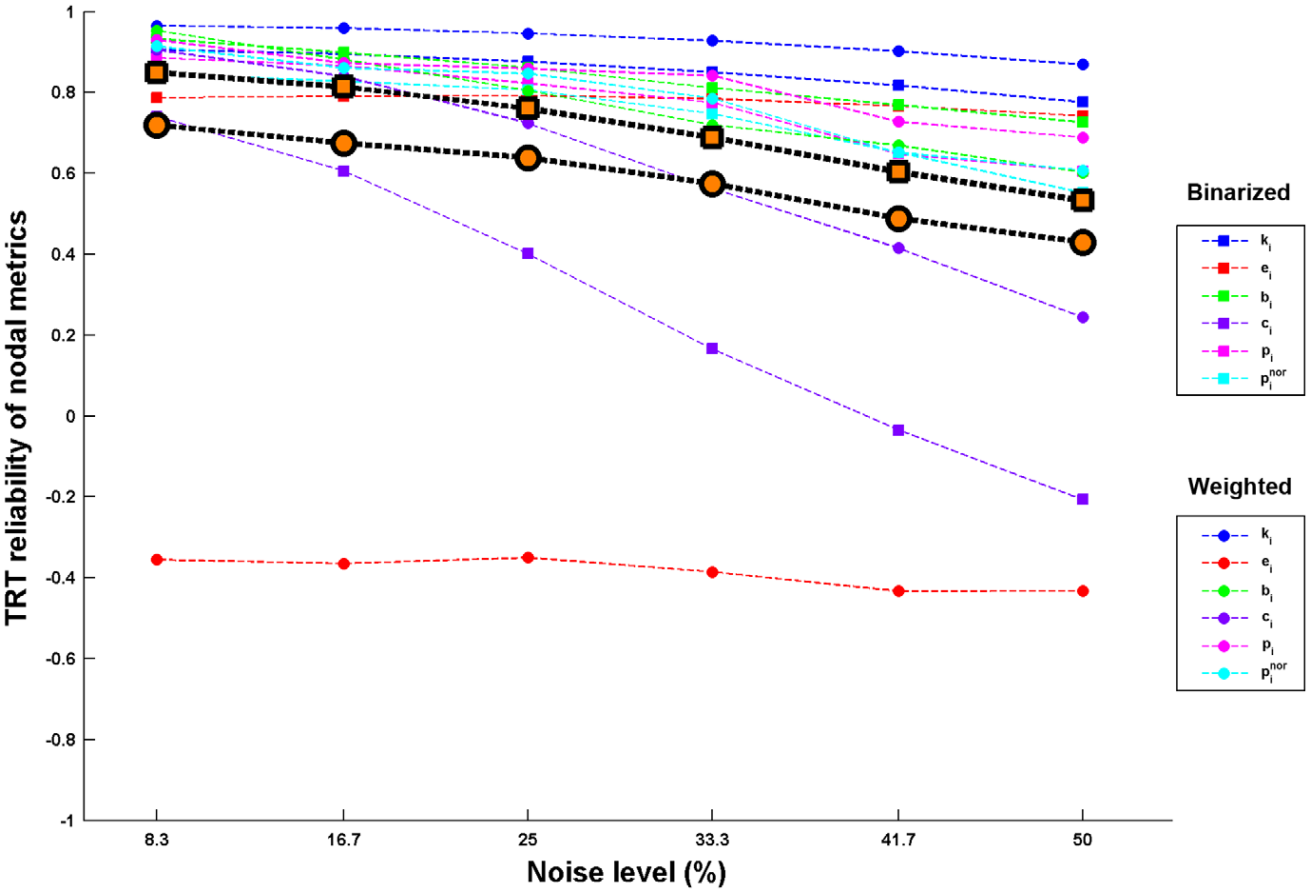
TRT reliability of nodal network metrics as a function of noise in RSFC for S-AAL-based networks. Nodal network metrics were sensitive to disturbances of RSFC and no differences were observed in the resistance to noise in functional connectivity between binarized and weighted network analysis. The highlighted black border marks are the average reliability across metrics for binarized (square) and weighted (circle) network analysis, respectively. Of note, although sensitive, nodal network metrics showed strong tolerance of disturbances in RSFC. TRT, test-retest; RSFC, resting-state functional connectivity.

By simulating functional connectivity matrices with different levels of noise, we found that: 1) for global network metrics, the TRT reliability was sensitive (F(5,55) = 23.303, p<10^-11^, repeated two-way ANOVA) to disturbances in functional connectivity values and weighted network analysis generated numerically more (F(1,11) = 5.183, p = 0.044, repeated two-way ANOVA) reliable results than binarized network analysis (Fig. 11); 2) for nodal network metrics, although sensitive to the levels of noise (F(5,25) = 7.762, p<10^−3^, repeated two-way ANOVA), they were highly resistant to numerical changes in functional connectivity and there were no differences (F(1,5) = 0.312, p = 0.601, repeated two-way ANOVA) in the resistance to noise between binarized and weighted network analyses (Fig. 12); 3) there were no differences in numerical stability against noise in functional connectivity (p>0.05 under each noise level) between the first-order and second-order network metrics (Table 2); 4) nodal network metrics were more numerically reliable than global network against noise in functional connectivity (p<10^−3^ under each noise level). Of note, although sensitive to functional connectivity noise, the degree varied dramatically among global metrics. For instance, small-world parameters and network efficiency were extremely sensitive to even little noise in functional connectivity while assortativity, hierarchy, synchronization and modularity were relatively resistant to noise (Fig. 11).

### TRT reliability of network metrics: S-HOA

#### Reliability of global network metrics

Analogous to results from S-AAL-based networks, S-HOA-based networks also showed overall low (Fig. S6a) but metric- (Fig. S6b) and threshold- (Fig. S7) sensitive reliability. However, unlike the finding of modest long-term reliability of multiple global metrics for S-AAL-based networks (Fig. 5 and Fig. 6a, left), S-HOA-based networks were mainly related with moderate short-term reliability in multiple global metrics (except for lambda) (Fig. S6a and Fig. S7). Of note, synchronization 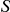 was found to repeatedly show overall moderate reliability (Fig. S6a). Subsequent statistical comparisons revealed that TRT reliability of global network metrics were modulated by NT factor (F(1,11) = 6.819, p = 0.024) with higher reliability observed for binarized networks (t(47) = 2.248, p = 0.029, paired t-test) (Table 3).

#### Reliability of local nodal metrics

Nodal reliability of S-HOA-based networks (Fig. S8) exhibited the same patterns as those for S-AAL-based networks of 1) factors independent (p>0.05 for all the factors of TI, NM and NT as well as all possible interactions) (Table 3), 2) metric-sensitive (F(5,35) = 12.098, p<10^−6^, degree was the most reliable and least variable) (Fig. S9), and 3) spatial heterogeneous distribution over the brain (Fig. S10a). The most reliable regions were also mainly unimodal and heteromodal association cortex regions and limbic/paralimbic regions of temporal and parietal lobes that were not replicated by other nodal metrics (Fig. S5b). Also, nodal centrality (Fig. S10b) showed no significant relationship (R^2^<2%) with nodal reliability (Fig. S10c and d).

### TRT reliability of network metrics: F-DOS

#### Reliability of global network metrics

**Figure 13:**
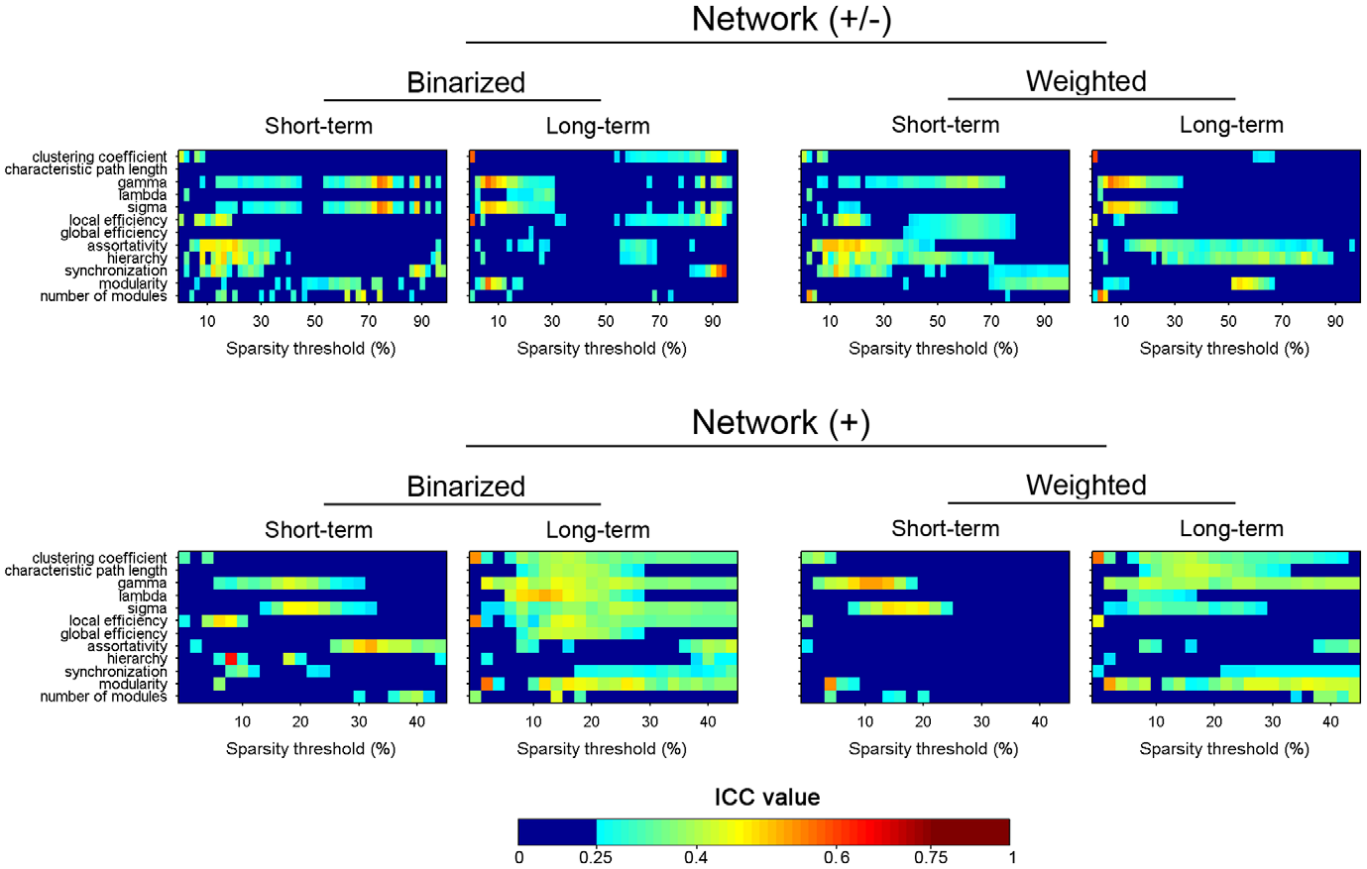
TRT reliability of global network metrics as a function of sparsity threshold for F-DOS-based networks. ICC values less than 0.25 were mapped to a single color of dark blue as well dark red color for ICC values greater than 0.75, respectively. Multiple network metrics showed modest reliability in certain threshold range. Network (+/-), networks constructed using absolute both positive and negative correlations; Network (+), networks constructed using only positive correlations; Binarized, binarized network anlysis; Weighted, weighted network analysis; TRT: test-retest; F-DOS, functional ROIs from Dosenbach et al. (2006, 2010).

In compared with structural ROIs-based networks, functional ROIs-based networks showed fair reliability in more global metrics over wider threshold range, especially for networks of positive correlations (Fig. 13). For example, small-world parameters (clustering coefficient 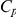, characteristic path length 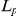, normalized clustering coefficient 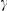, normalized characteristic path length 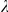 and small-worldness 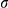) were fairly reliable (predominantly for long-term reliability) for positive networks. The threshold-independent reliability was presented in the right panel of Figure 6a. Subsequent statistical analyses revealed that, in contrast with the measure-related differences in global network reliability observed for structural ROIs based-networks (Fig. 6b, left and Fig. S6b), there was no significant differences (F(11,77) = 1.298, p = 0.242) among global metrics (Fig. 6b, right) for functional ROIs-based networks. Furthermore, unlike the sensitivity of global network reliability to experimental factor of TI and graph-based analytical strategies of NM and NT for structural ROIs-based networks, reliability of functional ROIs-based networks was robust against these factors (p>0.05) (Table 3).

#### Reliability of local nodal metrics

**Figure 14:**
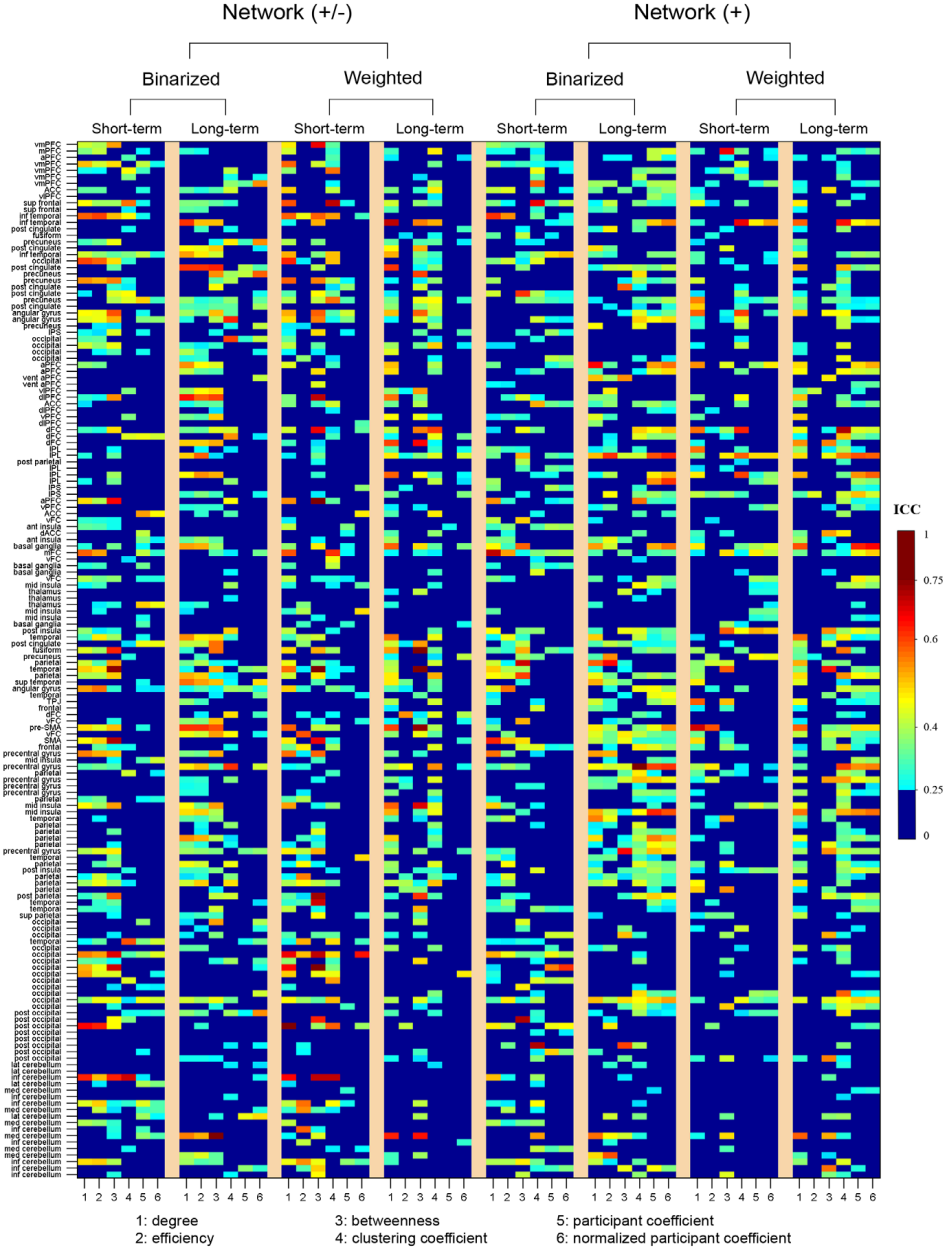
TRT reliability of nodal metrics for F-DOS-based networks. Nodal reliability varied across nodal attributes and spatial locations. The full names of region's abbreviations were listed as in Table S3. ICC values less than 0.25 were mapped to a single color of dark blue as well dark red color for ICC values greater than 0.75, respectively. Network (+/-), networks constructed using absolute both positive and negative correlations; Network (+), networks constructed using only positive correlations; Binarized, binarized network analysis; Weighted, weighted network analysis; TRT, test-retest; F-DOS, functional ROIs from Dosenbach et al. (2006, 2010).

**Figure 15:**
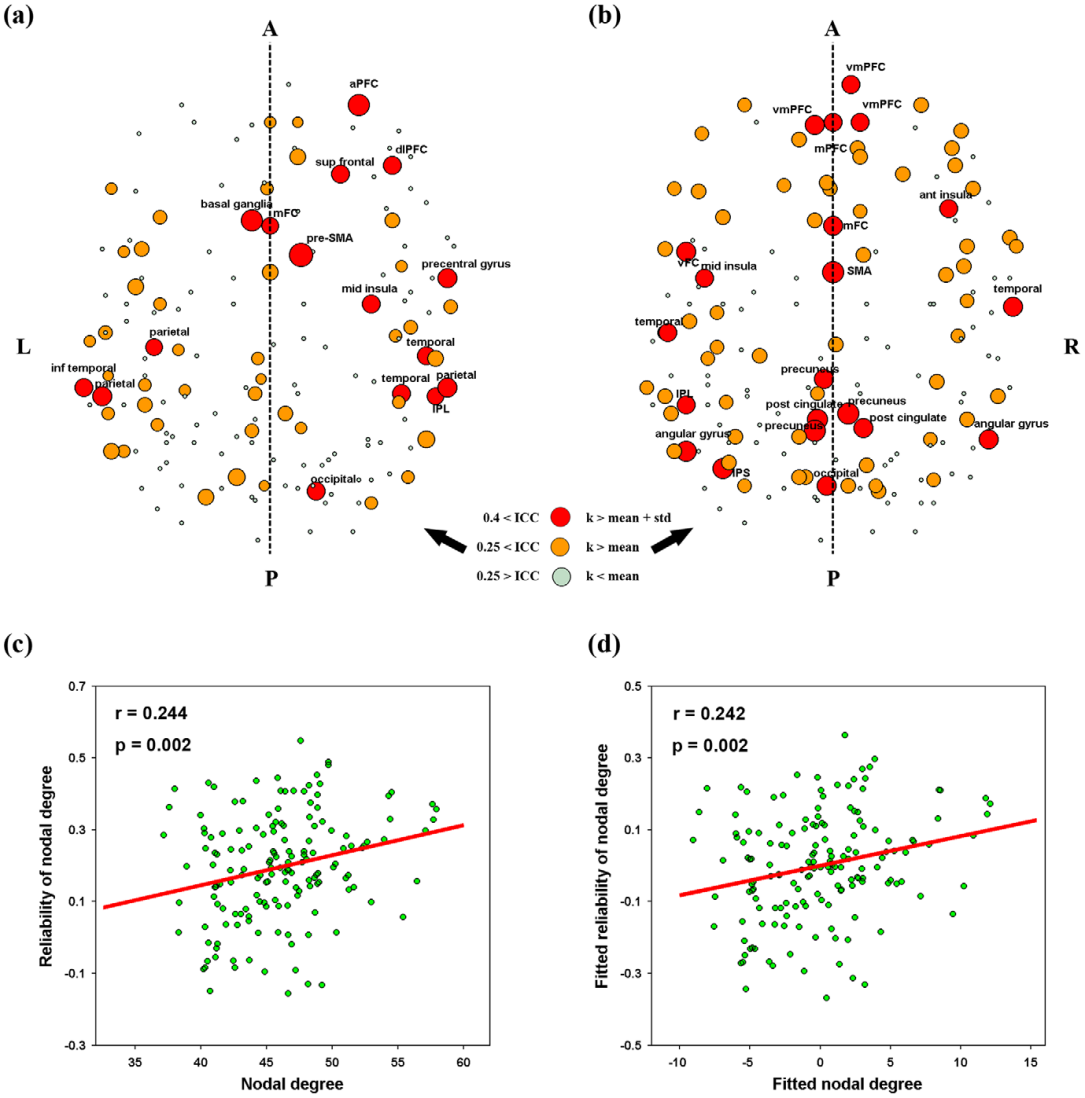
Nodal TRT reliability of degree and its relationship with nodal degree centrality for F-DOS-based networks. (a) Nodal TRT reliability was mapped in anatomical space after average across scanning time interval, network type and network membership because of no effects of these factors on nodal reliability. (b) Nodal degree centrality (AUCs) was also mapped in anatomical space which was averaged across subjects and factors of scanning time interval, network type and network membership. Trend lines were further obtained by linear least-square fit to reveal the relationship between nodal degree centrality and their corresponding reliability after with (d) and without (c) correcting for the effects of regional size. Of note, the full names of region's abbreviations were listed as in Table S1. TRT, test-retest; F-DOS, functional ROIs from Dosenbach et al. (2006, 2010); k, nodal degree; A, anterior; P, posterior; L, left; R, right.

Figure 14 delineated the nodal reliability for functional ROIs-based networks. No significant (p>0.05) effects were observed for TI, NM and NT on mean nodal reliability (Table 3), consistent with findings from structural ROIs-based networks (both S-AAL and S-HOA). Also analogous to findings of structural ROIs-based networks, nodal degree was found to show the highest reliability and least variance in compared with others (F(5,35) = 3.041, p = 0.022) (Fig. 8b). After averaged over factors of TI, NM and NT, mean nodal degree reliability showed that there were quite a few reliable regions distributed in bilateral temporal, parietal and the right frontal lobes (Fig. 15a). The nodal centrality pattern (Fig. 15b) can only explain a small fraction (R^2^<6%) of nodal reliability pattern (Fig. 15c and d). We also noted that the most reliable regions were predominantly located in the right hemisphere (Fig. 15a) and varied across nodal metrics (Fig. S5c).

## Discussion

In the present study, we examined the test-retest reliability of topological metrics of intrinsic connectivity networks derived from human brain R-fMRI data. First, we replicated previous findings that RSFC exhibited modest to high test-retest reliability [27]. Further reliability analyses of network metrics highlighted several main findings: 1) that global network metrics showed overall poor to low but threshold-sensitive reliability; 2) that local nodal metrics were fairly reliable for association and limbic/paralimbic cortex regions; 3) that reliability of network metrics (both global and local) differed significantly among the measures examined; 4) that reliability of global network metrics depended on multiple experiment and analytical factors while nodal reliability was robust to these factors; and 5) that weighted networks (compared to binarized networks) and nodal (compared to global) network metrics were numerically more reliable in the face of noise in functional connectivity. Taken together, we provided a systematically quantitative TRT reliability evaluation of topological metrics of R-fMRI based brain networks. Our findings suggested continued usage of graph theoretical approaches to explore brain networks and had potential relevance for guiding graph analytical schemes for R-fMRI to achieve reliable results.

For global network metrics, we observed overall low TRT reliability. This observation was consistent with results reported in a previous MEG study [12]. Indeed, compared with task engagement, Deuker et al. (2009) found that resting state was related with significantly lower reliability of global network metrics. This may be related to variable mental states of participants across scans which induce variations in RSFC [19], [20], [21], [22], [23], [24], [25]. Such discrepancies in RSFC especially in shortcuts or inter-module/component connections [54] may further affect the topological organization of the overall connectivity network [28]. To test the possibility, we examined the differences in RSFC strength between scans by paired t-tests. The results revealed that no connections showed significant differences (p<0.05, corrected) across scans, implying the temporal stability of RSFC [27], [29], [31]. Despite of non-significant differences, our simulation analysese indicated that global network metrics were extremely sensitive to numerical changes in RSFC, especially for small-world parameters and network efficiency. Another possible origin of low TRT reliability is due to low between-subject variance or low ability of global network metrics to differentiate subjects. With that said, the low TRT reliability of global metrics may suggest high consistency of global properties of intrinsic brain networks across subjects. Finally, the noise resulting from MRI data acquisition and coregistration inaccuracy may also influence network reliability, which should be elucidated in the future work.

Despite of the overall low TRT reliability, some global metrics showed relatively high reliability. For example, lambda showed moderate long-term reliability for structural both S-AAL and S-HOA-based networks. This may be due to the correction of absolute characteristic path length to referenced random networks which compensates for underlying differences of baseline networks. Further statistical analyses revealed significant differences in TRT reliability among global metrics, suggesting an obvious heterogeneity among different global network metrics in reliably capturing intrinsic brain architecture. Moreover, the profiles of global network reliability presented threshold sensitive patterns indicating the importance of threshold selection for reliable results. These findings raise the question of how to determine threshold for brain network studies. A compromise strategy is to investigate brain networks over a continuum threshold range under the circumstance that no sufficient knowledge exists for prior threshold selection.

Several factors were found to significantly affect the TRT reliability of global network metrics. First, inclusion of negative functional connectivity in brain networks tended to decrease TRT reliability of global network properties. Previous evidence has manifested that negative correlations showed greater population and state related variance in the spatial maps [55] and lower TRT reliability [27] relative to positive correlations. Consistent with these findings, our results suggest that negative connectivity should be treated with cautions for resting-state brain network studies, which may reduce the TRT reliability. It should be noted that the emergence of negative connectivity is related with the global signal regression, a currently controversial step in preprocessing R-fMRI data [56], [57], [58], [59].

Second, binarized networks outperformed weighted networks in TRT reliability of global network metrics. This finding seemed counterintuitive. Indeed, weighted networks could characterize network topology more precisely and detect more subtle network topological changes than binarized networks due to the consideration of connectivity strength [60]. However, this is not necessary to mean better reliability for weighted networks since the possibility that weighted networks may introduce simultaneously extra noise or overly model individual specific details. All these may lead to more within-subject variance (i.e., variance across scans) and thus lower reliability. Of note, our simulation results showed that weighted networks generated numerically more stable results against noise in functional connectivity in comparison to binarized networks. This suggests that the observed reliability derived from actual R-fMRI data were affected by various factors, not a single factor of numerical changes in functional connectivity.

Third, long-term scans showed higher TRT reliability of global network metrics than short-term scans. This finding was contrast to previous findings that RSFC exhibited higher TRT reliability for short-term interval scans [27]. It may reflect the fact that the average of scan2 and scan3 in the current study can potentially improve the estimation of long-term reliability, i.e., reduce within-session noise [29], [30]. To test this interpretation, we further calculated the long-term TRT reliability by using scan1 and scan3 and again found a long-term-larger-than-short-term pattern, indicating a robust finding. Nevertheless, further work is needed to verify this finding and aid in our understanding of how network topology interacts with the scanning procedure of time interval.

Finally, TRT reliability of global network metrics was modulated by strategies of network node definition. Specifically, reliability of only structural (S-AAL and S-HOA) rather than functional (F-DOS) ROIs-based networks depended on the factors of TI, NM and NT. The discrepancy may reflect different approaches of generating ROIs. Structural ROIs were obtained mainly in terms of anatomical features of sulcal pattern (S-AAL) [37] or standard anatomical boundaries (S-HOA) [38], [39] whereas functional ROIs were derived from previous meta-analyses of fMRI activation studies which carried specific functional information [40], [41]. Furthermore, even for structural ROIs based networks, the modulations of TI, NM and NT differed across parcellations. Previous studies have demonstrated that network properties were sensitive to nodal definition based on parcellation strategies [32], [36] and spatial scales [33], [34], [35]. Nevertheless, it's hard to conclude which approach or which parcellation is better since all of them are valid and important approaches to uncover brain connectivity architecture from different perspectives [40], [43], [61], [62]. Here, our results provide references for studying intrinsic brain networks, for example, binarized networks should be preferred for S-HOA-based intrinsic brain networks according to our results.

For local nodal metrics, nodal degree showed the highest reliability and least variance across factors of TI, NM and NT among the six nodal metrics. Using this metric, we found that some association cortex and limbic/paralimbic regions exhibited fair to good TRT reliability for S-AAL and S-HOA derived networks, such as precuneus, angular gyrus, superior forntal gurus, paracentral lobule, supramarginal gyrus, anterior cingulate gyrus, hippocampus and parahippocampal gyrus. Most of these regions have been identified to serve as structural or functional hubs/connectors in human brain networks [43], [44], [54], [60], [63], [64], [65], [66]. For F-DOS derived networks, more regions were modestly reliable, predominately located in the right frontal lobe and bilateral parietal and temporal lobes. Hubs are essential in supporting the performance of high cognitive functions of the human brain by integrating specialized brain regions into coordinated networks. Buckner and colleagues [64] demonstrated that the topography of human brain cortical hubs is highly similar across populations and robust against task states, therefore reflecting a stable property of brain functional architecture. Here, our results indicate that those reliable regions qualitatively tend to serve as hubs in intrinsic functional brain networks. Nonetheless, our quantitative analysis found that nodal reliability showed low correlations (although significant) with nodal centrality (R^2^<10%), suggesting limited predictive ability of nodal centrality on reliability. These findings imply that there may exist other factors affecting nodal reliability, such as the spatial locations of nodes or regions. Indeed, we found that posterior regions were more reliable than anterior regions even after correcting for the differences in functional connectivity across regions. This may reflect the nature of the brain in which the neural dynamics of spatially different brain regions are differently constrained in the resting-state. It would be an interesting question for future studies. Additionally, we noted that the most reliable regions appeared to predominately locate in the right hemisphere for F-DOS and S-HOA based networks. Hence, exploring brain functional asymmetry from the perspective of reliability may provide more insights into the brain's functional architecture.

Nodal reliability was found to be robust against the factors of TI, NM and NT, regardless of different node definition strategies. Simulation analyses revealed that nodal metrics were highly tolerant of fluctuations in functional connectivity values and were numerically more stable than global network metrics in the face of connectivity noise. The reliable and robust features of nodal metrics propose local nodal metrics as reliable candidates to reveal topological organization of intrinsic functional brain networks.

There are several issues that remained to be addressed in future. First, the reliability analyses of graph-based network metrics were conducted after several R-fMRI preprocessing steps. To date, how different preprocessing strategies affect the TRT reliability of network metrics is rarely investigated. Specifically, in the current study, RSFC were obtained based on band-pass filtered data (0.01–0.1 Hz). Previous R-fMRI studies have demonstrated frequency specific features for RSFC [67], [68], [69] and network topology [43], [46], [70], [71]. Accordingly, exploring the impacts of different preprocessing steps on TRT reliability, especially the filtering frequency bands, is an important topic to determine specific processing schemes for consistent, reliable results. Second, we limited our examination of TRT reliability to 12 global network properties and 6 nodal characteristics, which were widely used to characterize brain network architectures. However, there are still a lot of other network metrics, such as motif [72] and vulnerability [73] (for reviews, see [74], [75]) whose TRT reliability need to be evaluated in future. Finally, using R-fMRI, we examined the TRT reliability of intrinsic functional brain networks. Previous studies have performed similar analyses of structural or functional brain networks using DTI, MEG, or fMRI data during resting state or cognitive task engagement [10], [11], [12], [13]. Despite these advances, a systematic reliability evaluation using multimodal data from the same cohort of population is warranted to gain more insights into human brain's structural and functional architectures.

In conclusion, we studied the TRT reliability of graph-based network metrics derived from resting-state fMRI data and the effects of several factors on the reliability. Based on our findings, we provide some methodological recommendations for resting-state fMRI community in dealing with brain connectome studies. First, negative correlations need to be excluded or considered with cautions for S-AAL-based brain network studies. Second, binarized networks should be preferred for S-HOA-based brain network studies as compared to weighted networks. Third, reliability of functional ROIs-based networks was robust against the three factors of scanning time, network membership and network type. Finally, nodal metrics (especially nodal degree) could produce more reliable results and are more resilient to functional connectivity disturbances, which should be popularized in future brain network studies. Nonetheless, we pointed out that further work is necessary to standardize the methodological framework on this burgeoning field.

## Supporting Information

Figure S1 Spatial locations of functionally defined ROIs. These ROIs broadly but not completely cover the cerebral cortex and cerebellum without any overlap between ROIs and were associated with five functions of error-processing, default-mode, memory, language and sensorimotor. A, anterior; P, posterior; L, left; R, right.
(DOC)

Figure S2 Spatial similarity and TRT reliability patterns of S-HOA-based RSFC. Mean Pearson correlation matrices (a), consistency of overall patterns between mean matrices (b) and TRT reliability of individual connections as well as the relationship between short-term and long-term reliability (c) are illustrated. The mean correlation matrices exhibited high similarity from both visual inspection (a) and quantitative spatial correlation analyses (b). Further TRT reliability analyses revealed many connections exhibiting fair to excellent reliability (c, also see Fig. 2). Moreover, a significant (p<0.05) correlation was found in the ICC matrices between short-term and long-term scans (c). Functional connections linking inter-hemisphere homotopic regions, as highlighted by plus signs (+), showed high connectivity strength and many of them exhibited high reliability. TRT, test-retest; RSFC, resting-state functional connectivity; S-HOA, structural ROIs from Harvard-Oxford atlas. Of note, the structural ROIs were listed in the order as in Table S2.
(DOC)

Figure S3 Relationship between RSFC and TRT reliability for S-HOA-based correlation matrices. Scatter plots of mean connectivity strength against corresponding ICC values are depicted to show the relationship. The trend lines were obtained by linear least-square fit. Significant (p<0.05) positive correlations were found between positive RSFC and their corresponding ICC values for both short-term and long-term scanning. In addition, significant negative correlations were also found for negative RSFC with their corresponding ICC values but only for long-term scanning. These findings suggest higher reliability for stronger RSFC. Functional connections linking inter-hemisphere homotopic regions are highlighted by plus signs (+). RSFC, resting-state functional connectivity; TRT, test-retest; S-HOA, structural ROIs from Harvard-Oxford atlas.
(DOC)

Figure S4 The absolute correlation thresholds under each sparsity level for all the three sets of ROIs based networks. The correlation thresholds decrease with the increase of sparsity and are comparable across scans and across subjects for each set of ROIs-based networks. Of note, negative correlations were included.
(DOC)

Figure S5 Ranks of reliable regions revealed by nodal degree over other nodal metrics. (a) S-AAL-based networks; (b), S-HOA-based networks; (c) F-DOS-based networks. The ranks of those most reliable regions in terms of nodal degree (regions with ICC>0.4 in Fig. 9a, Fig. S10a and Fig. 15a) changed dramatically over nodal metrics for all ROIs sets, indicating inconsistency for most reliable regions. The full names of region's abbreviations were listed as in Table S1, S2 and S3.
(DOC)

Figure S6 TRT reliability of summarized global network metrics (a) and metric-related differences in reliability (b). The area under curve (AUC) of each metric was used to provide threshold-independent reliability estimation. Different metrics showed variable levels of reliability. Several of them were moderately reliable (e.g., lambda and synchronization). Subsequent statistical analysis revealed significant differences in TRT reliability among the 12 global network metrics, with lambda showing relatively high reliability and low variance. ICC values less than 0.25 were mapped to a single color of dark blue as well dark red color for ICC values greater than 0.75, respectively in (a). Network (+/-), networks constructed using absolute both positive and negative correlations; Network (+), networks constructed using only positive correlations; Binarized, binarized network analysis; Weighted, weighted network analysis; TRT, test-retest; S-HOA, structural ROIs from Harvard-Oxford atlas.
(DOC)

Figure S7 TRT reliability of global network metrics as a function of sparsity threshold for S-HOA-based networks. ICC values less than 0.25 were mapped to a single color of dark blue as well dark red color for ICC values greater than 0.75, respectively. Network (+/-), networks constructed using absolute both positive and negative correlations; Network (+), networks constructed using only positive correlations; Binarized, binarized network anlysis; Weighted, weighted network analysis; TRT: test-retest; S-HOA, structural ROIs from Harvard-Oxford atlas.
(DOC)

Figure S8 TRT reliability of nodal metrics for S-HOA-based networks. Nodal reliability varied across nodal attributes and spatial locations. Moreover, removing negative correlations seemed to result in more regions showing higher reliability in more nodal attributes (predominantly for binarized networks). The full names of region's abbreviations were listed as in Table S2. ICC values less than 0.25 were mapped to a single color of dark blue as well dark red color for ICC values greater than 0.75, respectively. Network (+/-), networks constructed using absolute both positive and negative correlations; Network (+), networks constructed using only positive correlations; Binarized, binarized network analysis; Weighted, weighted network analysis; TRT, test-retest; S-HOA, structural ROIs from Harvard-Oxford atlas.
(DOC)

Figure S9 Boxplot of mean nodal TRT reliability for S-HOA-based networks. Significant differences were found in the mean nodal reliability among the six nodal metrics examined with nodal degree showing the highest ICC values and least variances. TRT, test-retest; S-HOA, structural ROIs from Harvard-Oxford atlas.
(DOC)

Figure S10 **N**odal TRT reliability of degree and its relationship with nodal degree centrality for S-HOA-based networks. (a) Nodal TRT reliability was mapped in anatomical space after average across scanning time interval, network type and network membership because of no effects of these factors on nodal reliability. (b) Nodal degree centrality (AUCs) was also mapped in anatomical space which was averaged across subjects and factors of scanning time interval, network type and network membership. Trend lines were further obtained by linear least-square fit to reveal the relationship between nodal degree centrality and their corresponding reliability after with (d) and without (c) correcting for the effects of regional size. Of note, the full names of region's abbreviations were listed as in Table S2. TRT, test-retest; S-HOA, structural ROIs from Harvard-Oxford atlas; k, nodal degree; A, anterior; P, posterior; L, left; R, right.
(DOC)

Table S1 Regions of interest from S-AAL.
(DOC)

Table S2 Regions of interest from S-HOA.
(DOC)

Table S3 Regions of interest from F-DOS.
(DOC)

Table S4 Correlation coefficients between long-term reliability estimated by scan1 and the average of scan 2 and scan 3 and those estimated by scan 1 and scan 3 alone.
(DOC)

Text S1 Mathematical definitions of network metrics.
(DOC)
