# Supplementary material for: Graph Theoretical Analysis of Functional Brain Networks: Test-Retest Evaluation on Short- and Long-Term Resting-State Functional MRI Data
Source: PLoS One. 2011 Jul 19;6(7):e21976. doi: 10.1371/journal.pone.0021976 (PMC3139595; doi:10.1371/journal.pone.0021976)
Supplement: Figure S1 — Spatial locations of functionally defined ROIs. These ROIs broadly but not completely cover the cerebral cortex and cerebellum without any overlap between ROIs and were associated with five functions of error-processing, default-mode, memory, language and sensorimotor. A, anterior; P, posterior; L, left; R, right. (DOC) [file pone.0021976.s001.doc]

**Supporting Figure S1.** Spatial locations of functionally defined ROIs. These ROIs broadly but not completely cover the cerebral cortex and cerebellum without any overlap between ROIs and were associated with five different kinds of brain functions of error-processing, default-mode, memory, language and sensorimotor. Of note, several ROIs of cerebellum are outside of the brain surface template which does not contain cerebellum. A, anterior; P, posterior; L, left; R, right.


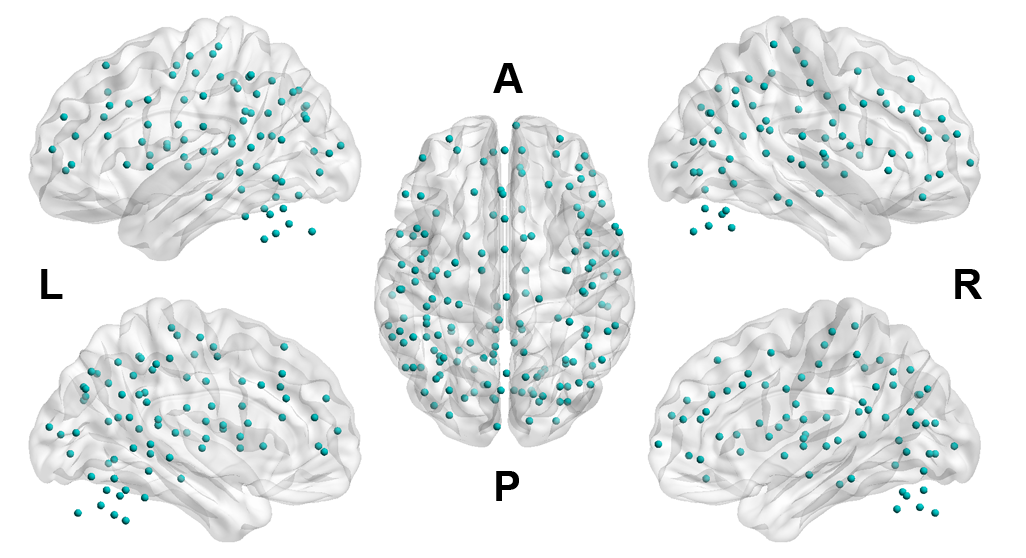


**Figure S1.** Spatial locations of functionally defined ROIs
