# Supplementary material for: Graph Theoretical Analysis of Functional Brain Networks: Test-Retest Evaluation on Short- and Long-Term Resting-State Functional MRI Data
Source: PLoS One. 2011 Jul 19;6(7):e21976. doi: 10.1371/journal.pone.0021976 (PMC3139595; doi:10.1371/journal.pone.0021976)
Supplement: Figure S2 — Spatial similarity and TRT reliability patterns of S-HOA-based RSFC. Mean Pearson correlation matrices (a), consistency of overall patterns between mean matrices (b) and TRT reliability of individual connections as well as the relationship between short-term and long-term reliability (c) are illustrated. The mean correlation matrices exhibited high similarity from both visual inspection (a) and quantitative spatial correlation analyses (b). Further TRT reliability analyses revealed many connections exhibiting fair to excellent reliability (c, also see Fig. 2). Moreover, a significant (p<0.05) correlation was found in the ICC matrices between short-term and long-term scans (c). Functional connections linking inter-hemisphere homotopic regions, as highlighted by plus signs (+), showed high connectivity strength and many of them exhibited high reliability. TRT, test-retest; RSFC, resting-state functional connectivity; S-HOA, structural ROIs from Harvard-Oxford atlas. Of note, the structural ROIs were listed in the order as in Table S2. (DOC) [file pone.0021976.s002.doc]

**Supporting Figure S2.** Spatial similarity and TRT reliability patterns of S-HOA-based RSFC. Mean Pearson correlation matrices (a), consistency of overall patterns between mean matrices (b) and TRT reliability of individual connections as well as the relationship between short-term and long-term reliability (c) are illustrated. The mean correlation matrices exhibited high similarity from both visual inspection (a) and quantitative spatial correlation analyses (b). Further TRT reliability analyses revealed many connections exhibiting fair to excellent reliability (c, also see Fig. 2). Moreover, a significant (p < 0.05) correlation was found in the ICC matrices between short-term and long-term scans (c). Functional connections linking inter-hemisphere homotopic regions, as highlighted by plus signs (+), showed high connectivity strength and many of them exhibited high reliability. TRT, test-retest; RSFC, resting-state functional connectivity; S-HOA, structural ROIs from Harvard-Oxford atlas. Of note, the structural ROIs were listed in the order as in Table S2.


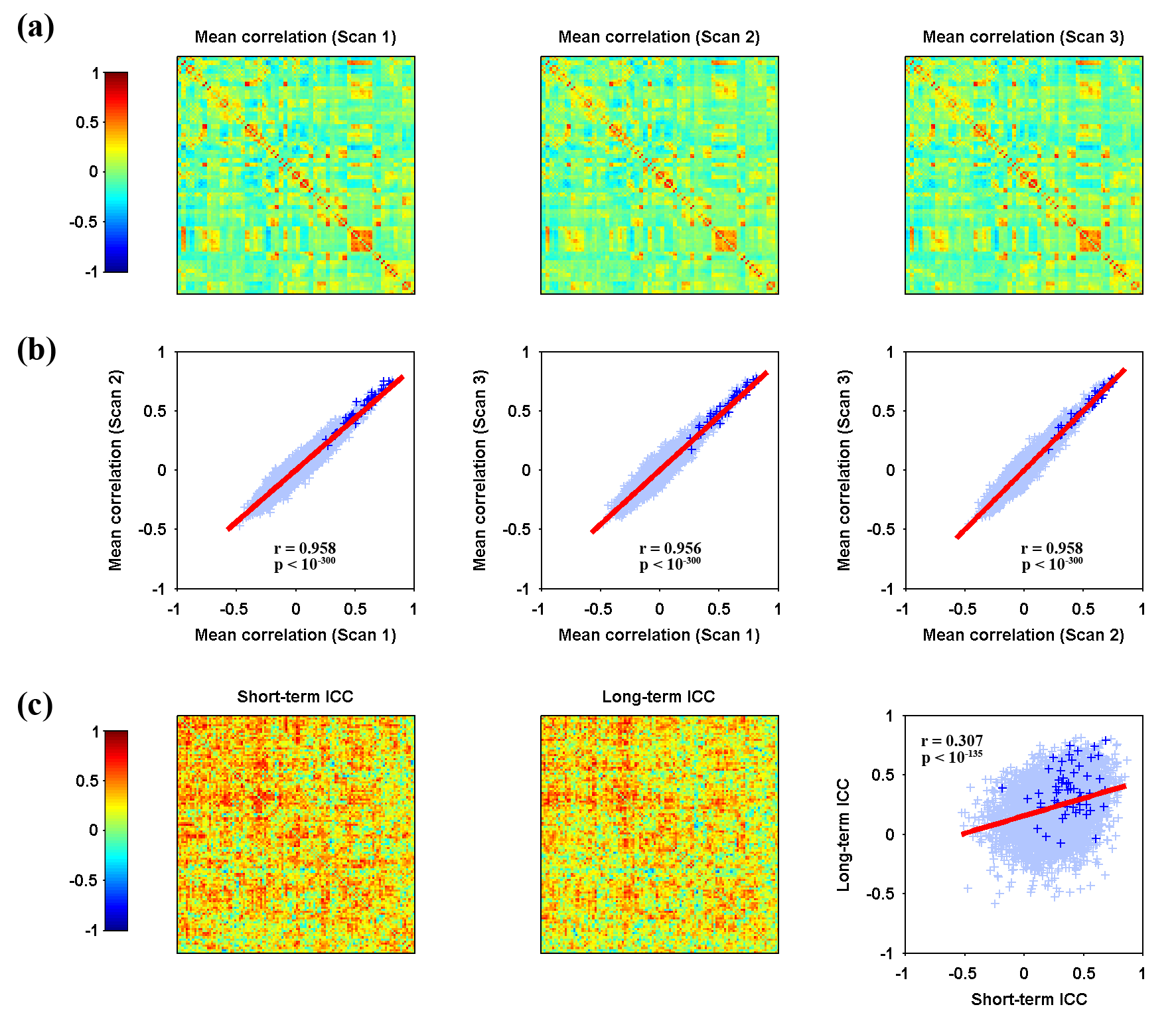


**Figure S2.** Spatial similarity and TRT reliability patterns of S-HOA-based RSFC
