# Supplementary material for: Graph Theoretical Analysis of Functional Brain Networks: Test-Retest Evaluation on Short- and Long-Term Resting-State Functional MRI Data
Source: PLoS One. 2011 Jul 19;6(7):e21976. doi: 10.1371/journal.pone.0021976 (PMC3139595; doi:10.1371/journal.pone.0021976)
Supplement: Figure S3 — Relationship between RSFC and TRT reliability for S-HOA-based correlation matrices. Scatter plots of mean connectivity strength against corresponding ICC values are depicted to show the relationship. The trend lines were obtained by linear least-square fit. Significant (p<0.05) positive correlations were found between positive RSFC and their corresponding ICC values for both short-term and long-term scanning. In addition, significant negative correlations were also found for negative RSFC with their corresponding ICC values but only for long-term scanning. These findings suggest higher reliability for stronger RSFC. Functional connections linking inter-hemisphere homotopic regions are highlighted by plus signs (+). RSFC, resting-state functional connectivity; TRT, test-retest; S-HOA, structural ROIs from Harvard-Oxford atlas. (DOC) [file pone.0021976.s003.doc]

**Supporting Figure S3.** Relationship between RSFC and TRT reliability for S-HOA-based correlation matrices. Scatter plots of mean connectivity strength against corresponding ICC values are depicted. The trend lines were obtained by linear least-square fit. Significant (p < 0.05) positive correlations were found between positive RSFC and their corresponding ICC values for both short-term and long-term scanning. In addition, significant negative correlations were also found for negative RSFC with their corresponding ICC values but only for long-term scanning. These findings suggest higher reliability for stronger RSFC. Functional connections linking inter-hemisphere homotopic regions are highlighted by plus signs (+). RSFC, resting-state functional connectivity; TRT, test-retest; S-HOA, structural ROIs from Harvard-Oxford atlas.


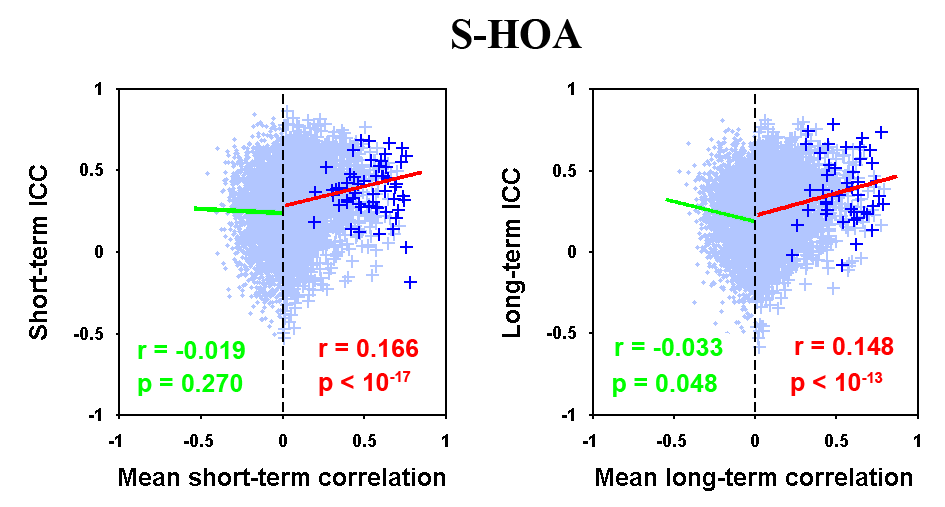


**Figure S3.** Relationship between RSFC and TRT reliability for S-HOA-based correlation matrices
