# Supplementary material for: Graph Theoretical Analysis of Functional Brain Networks: Test-Retest Evaluation on Short- and Long-Term Resting-State Functional MRI Data
Source: PLoS One. 2011 Jul 19;6(7):e21976. doi: 10.1371/journal.pone.0021976 (PMC3139595; doi:10.1371/journal.pone.0021976)
Supplement: Figure S4 — The absolute correlation thresholds under each sparsity level for all the three sets of ROIs based networks. The correlation thresholds decrease with the increase of sparsity and are comparable across scans and across subjects for each set of ROIs-based networks. Of note, negative correlations were included. (DOC) [file pone.0021976.s004.doc]

**Supporting Figure S4.** The absolute correlation thresholds under each sparsity level for all the three sets of ROIs based networks. The correlation thresholds decrease with the increase of sparsity and are comparable across scans and across subjects for each set of ROIs-based networks. Of note, negative correlations were included. Error bar indicates standard deviation of the mean across subjects under each scan (upper row). The standard deviation across scans for each subject is not shown (bottom row) for the sake of conciseness.


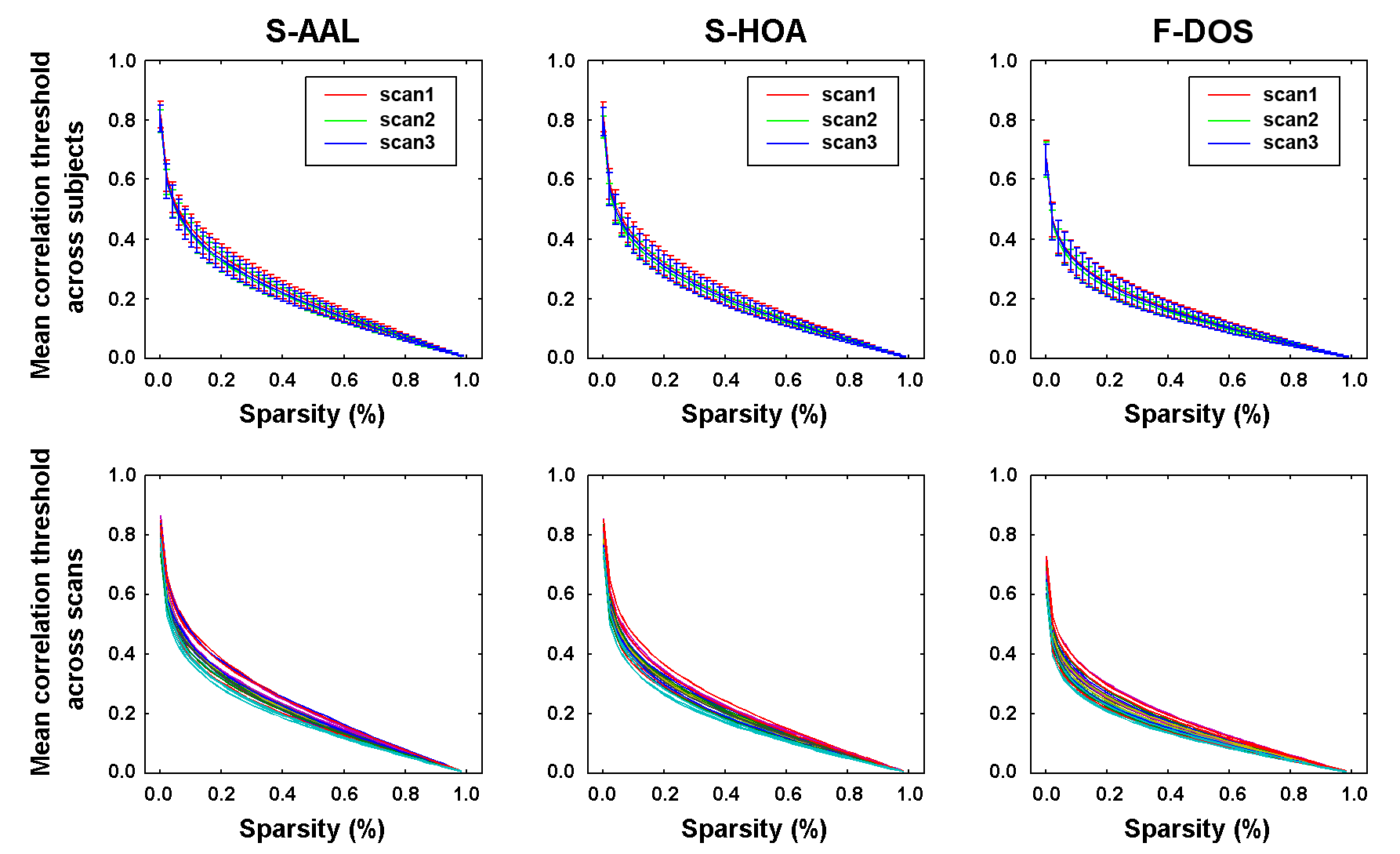


**Figure S4.** The absolute correlation thresholds under each sparsity level for all the three sets of ROIs based networks
