# Supplementary material for: Graph Theoretical Analysis of Functional Brain Networks: Test-Retest Evaluation on Short- and Long-Term Resting-State Functional MRI Data
Source: PLoS One. 2011 Jul 19;6(7):e21976. doi: 10.1371/journal.pone.0021976 (PMC3139595; doi:10.1371/journal.pone.0021976)
Supplement: Figure S5 — Ranks of reliable regions revealed by nodal degree over other nodal metrics. (a) S-AAL-based networks; (b), S-HOA-based networks; (c) F-DOS-based networks. The ranks of those most reliable regions in terms of nodal degree (regions with ICC>0.4 in Fig. 9a, Fig. S10a and Fig. 15a) changed dramatically over nodal metrics for all ROIs sets, indicating inconsistency for most reliable regions. The full names of region's abbreviations were listed as in Table S1, S2 and S3. (DOC) [file pone.0021976.s005.doc]

**Supporting Figure S5.** Ranks of reliable regions revealed by nodal degree over other nodal metrics. (a) S-AAL-based networks; (b), S-HOA-based networks; (c) F-DOS-based networks. The ranks of those most reliable regions in terms of nodal degree (regions with ICC > 0.4 in Fig. 9a, Fig. S10a and Fig. 15a) changed dramatically over nodal metrics for all ROIs sets, indicating inconsistency for most reliable regions. The full names of region’s abbreviations were listed as in Table S1, S2 and S3.


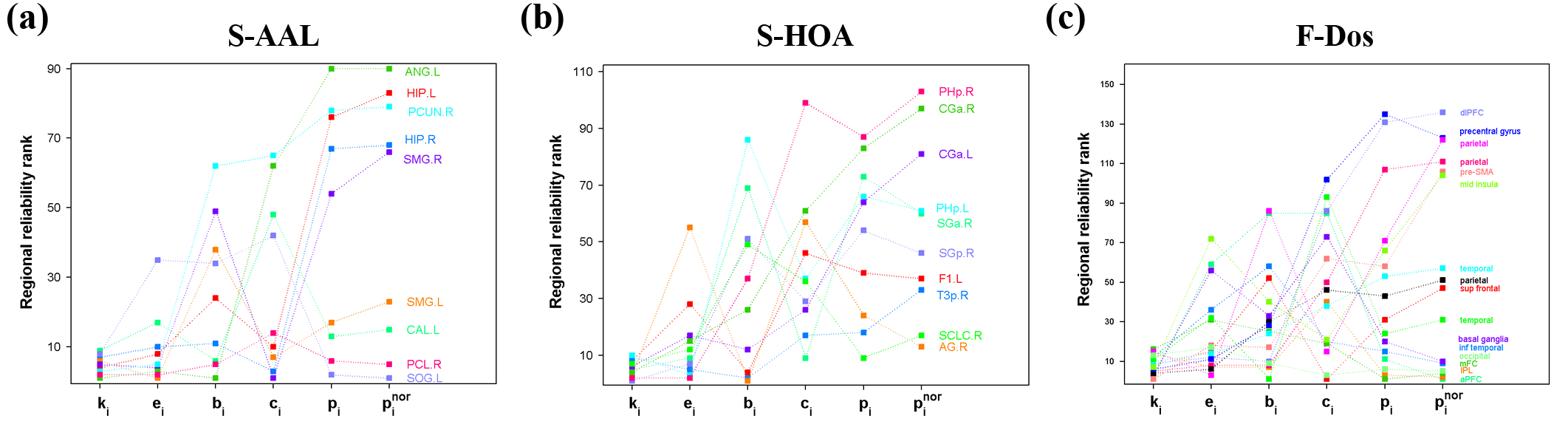


**Figure S5.** Ranks of reliable regions revealed by nodal degree over other nodal metrics
