# Supplementary material for: Graph Theoretical Analysis of Functional Brain Networks: Test-Retest Evaluation on Short- and Long-Term Resting-State Functional MRI Data
Source: PLoS One. 2011 Jul 19;6(7):e21976. doi: 10.1371/journal.pone.0021976 (PMC3139595; doi:10.1371/journal.pone.0021976)
Supplement: Figure S6 — TRT reliability of summarized global network metrics (a) and metric-related differences in reliability (b). The area under curve (AUC) of each metric was used to provide threshold-independent reliability estimation. Different metrics showed variable levels of reliability. Several of them were moderately reliable (e.g., lambda and synchronization). Subsequent statistical analysis revealed significant differences in TRT reliability among the 12 global network metrics, with lambda showing relatively high reliability and low variance. ICC values less than 0.25 were mapped to a single color of dark blue as well dark red color for ICC values greater than 0.75, respectively in (a). Network (+/-), networks constructed using absolute both positive and negative correlations; Network (+), networks constructed using only positive correlations; Binarized, binarized network analysis; Weighted, weighted network analysis; TRT, test-retest; S-HOA, structural ROIs from Harvard-Oxford atlas. (DOC) [file pone.0021976.s006.doc]

**Supporting Figure S6.** TRT reliability of summarized global network metrics (a) and metric-related differences in reliability (b). The area under curve (AUC) of each metric was used to provide threshold-independent reliability estimation. Different metrics showed variable levels of reliability. Several of them were moderately reliable (e.g., lambda and synchronization). Subsequent statistical analysis revealed significant differences in TRT reliability among the 12 global network metrics, with lambda showing relatively high reliability and low variance. ICC values less than 0.25 were mapped to a single color of dark blue as well dark red color for ICC values greater than 0.75, respectively in (a). Network (+/-), networks constructed using absolute both positive and negative correlations; Network (+), networks constructed using only positive correlations; Binarized, binarized network analysis; Weighted, weighted network analysis; TRT, test-retest; S-HOA, structural ROIs from Harvard-Oxford atlas.


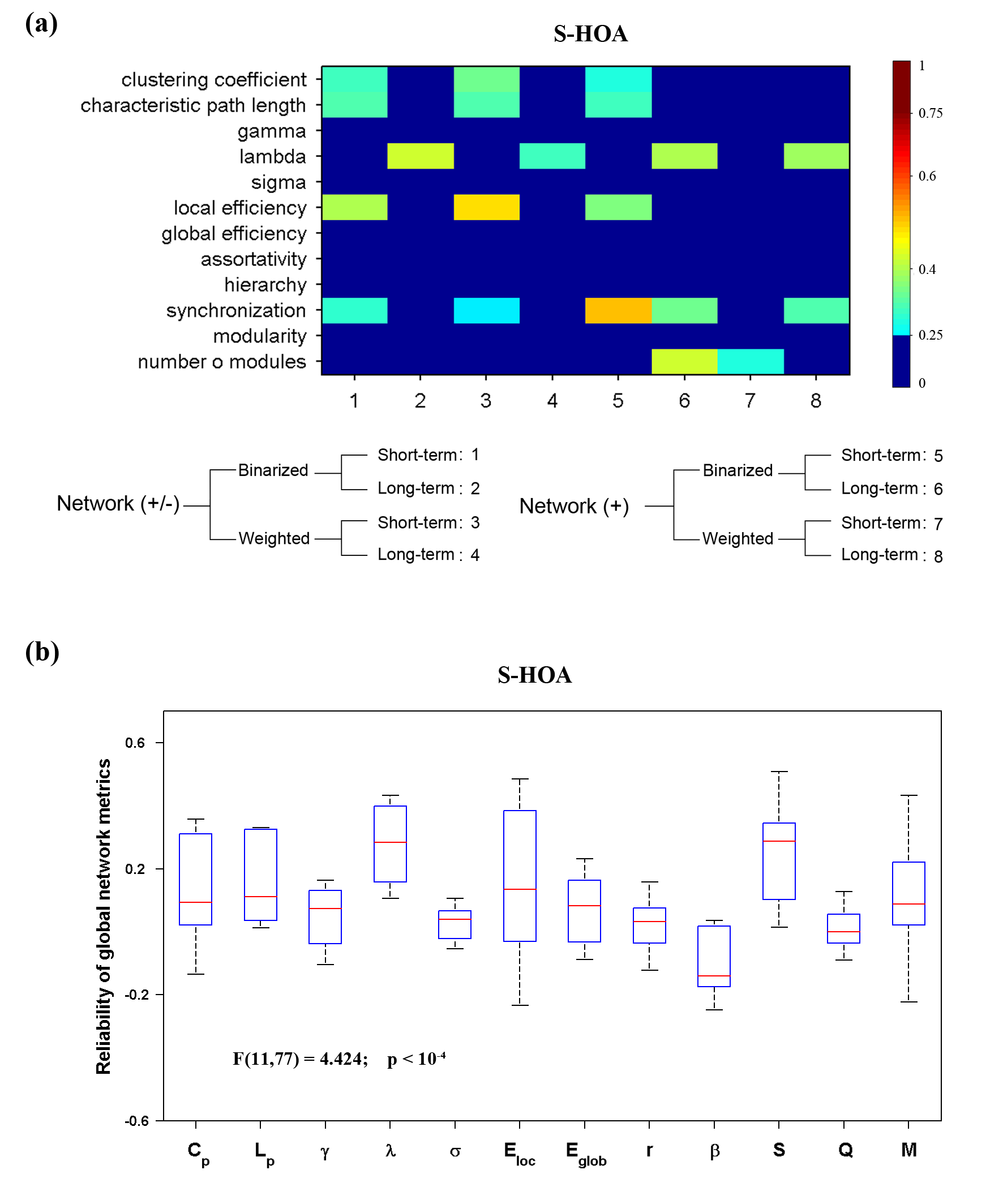


**Figure S6.** Threshold-independent TRT reliability of global network metrics (a) and their metric-related differences (b) for S-HOA-based networks
