# Supplementary material for: Graph Theoretical Analysis of Functional Brain Networks: Test-Retest Evaluation on Short- and Long-Term Resting-State Functional MRI Data
Source: PLoS One. 2011 Jul 19;6(7):e21976. doi: 10.1371/journal.pone.0021976 (PMC3139595; doi:10.1371/journal.pone.0021976)
Supplement: Figure S7 — TRT reliability of global network metrics as a function of sparsity threshold for S-HOA-based networks. ICC values less than 0.25 were mapped to a single color of dark blue as well dark red color for ICC values greater than 0.75, respectively. Network (+/-), networks constructed using absolute both positive and negative correlations; Network (+), networks constructed using only positive correlations; Binarized, binarized network anlysis; Weighted, weighted network analysis; TRT: test-retest; S-HOA, structural ROIs from Harvard-Oxford atlas. (DOC) [file pone.0021976.s007.doc]

**Supporting Figure S7.** TRT reliability of global network metrics as a function of sparsity threshold for S-HOA-based networks. ICC values less than 0.25 were mapped to a single color of dark blue as well dark red color for ICC values greater than 0.75, respectively. Network (+/-), networks constructed using absolute both positive and negative correlations; Network (+), networks constructed using only positive correlations; Binarized, binarized network anlysis; Weighted, weighted network analysis; TRT: test-retest; S-HOA, structural ROIs from Harvard-Oxford atlas.


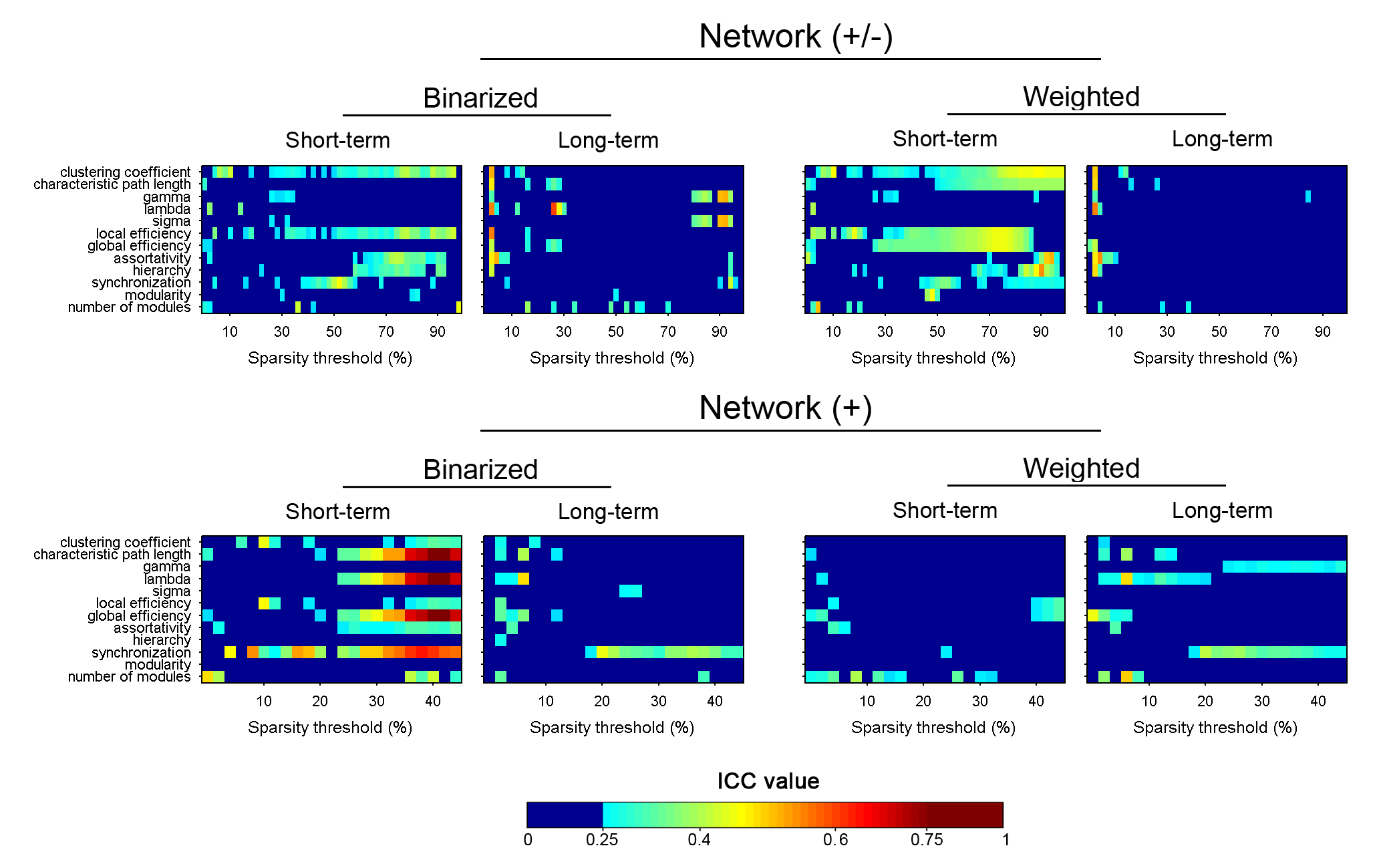


**Figure S7.** TRT reliability of global network metrics as a function of sparsity threshold for S-HOA-based networks
