# Supplementary material for: Graph Theoretical Analysis of Functional Brain Networks: Test-Retest Evaluation on Short- and Long-Term Resting-State Functional MRI Data
Source: PLoS One. 2011 Jul 19;6(7):e21976. doi: 10.1371/journal.pone.0021976 (PMC3139595; doi:10.1371/journal.pone.0021976)
Supplement: Figure S8 — TRT reliability of nodal metrics for S-HOA-based networks. Nodal reliability varied across nodal attributes and spatial locations. Moreover, removing negative correlations seemed to result in more regions showing higher reliability in more nodal attributes (predominantly for binarized networks). The full names of region's abbreviations were listed as in Table S2. ICC values less than 0.25 were mapped to a single color of dark blue as well dark red color for ICC values greater than 0.75, respectively. Network (+/-), networks constructed using absolute both positive and negative correlations; Network (+), networks constructed using only positive correlations; Binarized, binarized network analysis; Weighted, weighted network analysis; TRT, test-retest; S-HOA, structural ROIs from Harvard-Oxford atlas. (DOC) [file pone.0021976.s008.doc]

**Supporting Figure S8.** TRT reliability of nodal metrics for S-HOA-based networks. Nodal reliability varied across nodal attributes and spatial locations. Moreover, removing negative correlations seemed to result in more regions showing higher reliability in more nodal attributes (predominantly for binarized networks). The full names of region’s abbreviations were listed as in Table S2. ICC values less than 0.25 were mapped to a single color of dark blue as well dark red color for ICC values greater than 0.75, respectively. Network (+/-), networks constructed using absolute both positive and negative correlations; Network (+), networks constructed using only positive correlations; Binarized, binarized network analysis; Weighted, weighted network analysis; TRT, test-retest; S-HOA, structural ROIs from Harvard-Oxford atlas.


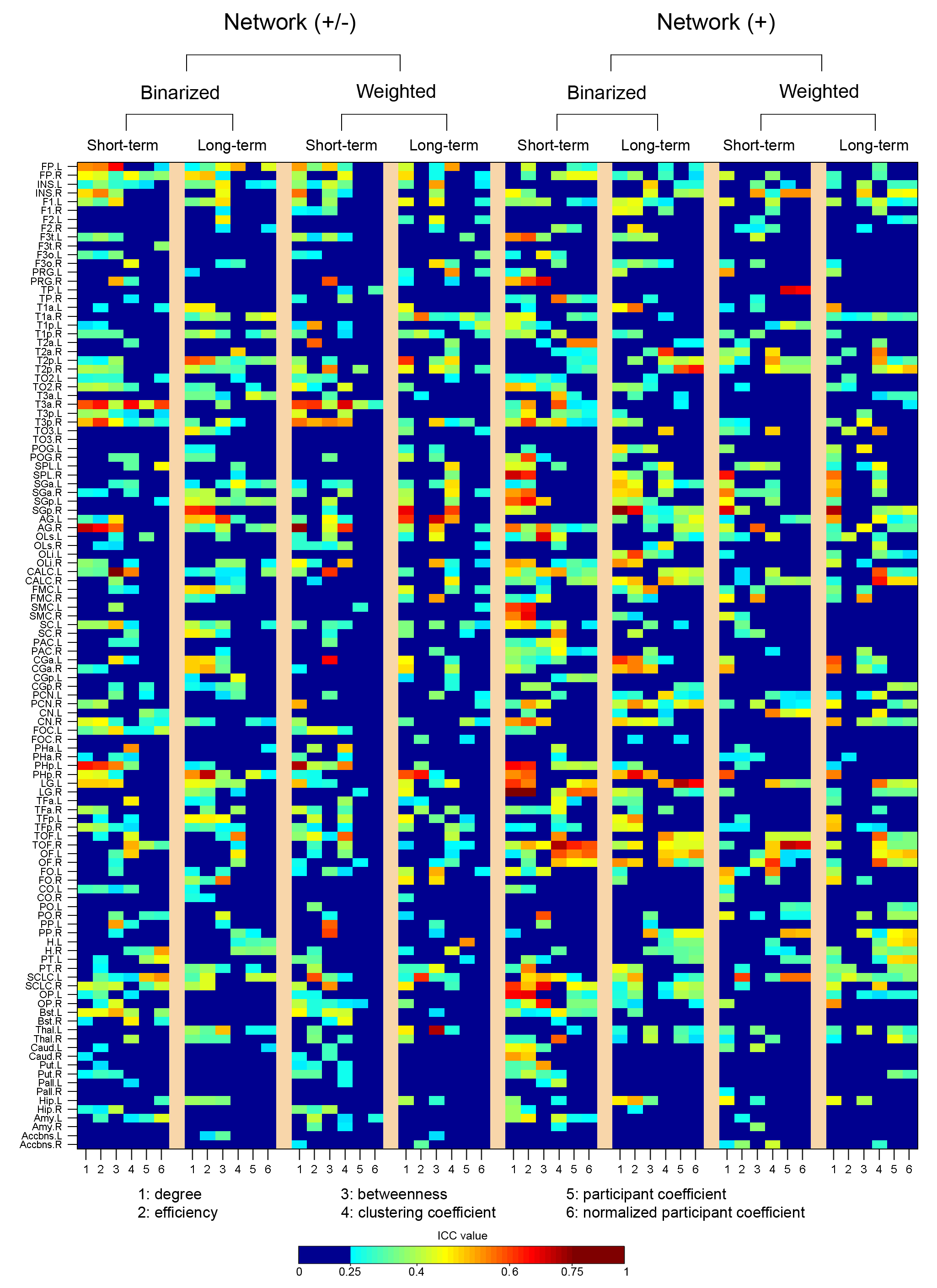


**Figure S8.** TRT reliability of nodal metrics for S-HOA-based networks
