# Supplementary material for: Graph Theoretical Analysis of Functional Brain Networks: Test-Retest Evaluation on Short- and Long-Term Resting-State Functional MRI Data
Source: PLoS One. 2011 Jul 19;6(7):e21976. doi: 10.1371/journal.pone.0021976 (PMC3139595; doi:10.1371/journal.pone.0021976)
Supplement: Figure S9 — Boxplot of mean nodal TRT reliability for S-HOA-based networks. Significant differences were found in the mean nodal reliability among the six nodal metrics examined with nodal degree showing the highest ICC values and least variances. TRT, test-retest; S-HOA, structural ROIs from Harvard-Oxford atlas. (DOC) [file pone.0021976.s009.doc]

**Supporting Figure S9.** Boxplot of mean nodal TRT reliability for S-HOA-based networks. Significant differences were found in the mean nodal reliability among the six nodal metrics examined with nodal degree showing the highest ICC values and least variances. TRT, test-retest; S-HOA, structural ROIs from Harvard-Oxford atlas.


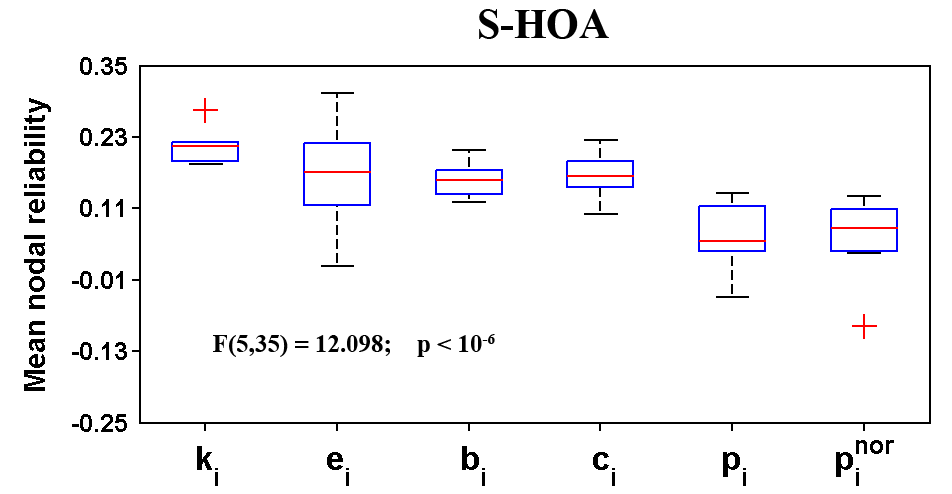


**Figure S9.** Boxplot of mean nodal TRT reliability for S-HOA-based networks
