# Supplementary material for: Graph Theoretical Analysis of Functional Brain Networks: Test-Retest Evaluation on Short- and Long-Term Resting-State Functional MRI Data
Source: PLoS One. 2011 Jul 19;6(7):e21976. doi: 10.1371/journal.pone.0021976 (PMC3139595; doi:10.1371/journal.pone.0021976)
Supplement: Figure S10 — Nodal TRT reliability of degree and its relationship with nodal degree centrality for S-HOA-based networks. (a) Nodal TRT reliability was mapped in anatomical space after average across scanning time interval, network type and network membership because of no effects of these factors on nodal reliability. (b) Nodal degree centrality (AUCs) was also mapped in anatomical space which was averaged across subjects and factors of scanning time interval, network type and network membership. Trend lines were further obtained by linear least-square fit to reveal the relationship between nodal degree centrality and their corresponding reliability after with (d) and without (c) correcting for the effects of regional size. Of note, the full names of region's abbreviations were listed as in Table S2. TRT, test-retest; S-HOA, structural ROIs from Harvard-Oxford atlas; k, nodal degree; A, anterior; P, posterior; L, left; R, right. (DOC) [file pone.0021976.s010.doc]

**Supporting Figure S10.** Nodal TRT reliability of degree and its relationship with nodal degree centrality for S-HOA-based networks. (a) Nodal TRT reliability was mapped in anatomical space after average across scanning time interval, network type and network membership because of no effects of these factors on nodal reliability. (b) Nodal degree centrality (AUCs) was also mapped in anatomical space, which was averaged across subjects and factors of scanning time interval, network type and network membership. Trend lines were further obtained by linear least-square fit to reveal the relationship between nodal degree centrality and their corresponding reliability after with (d) and without (c) correcting for the effects of regional size. Of note, the full names of region’s abbreviations were listed as in Table S2. TRT, test-retest; S-HOA, structural ROIs from Harvard-Oxford atlas; k, nodal degree; A, anterior; P, posterior; L, left; R, right.


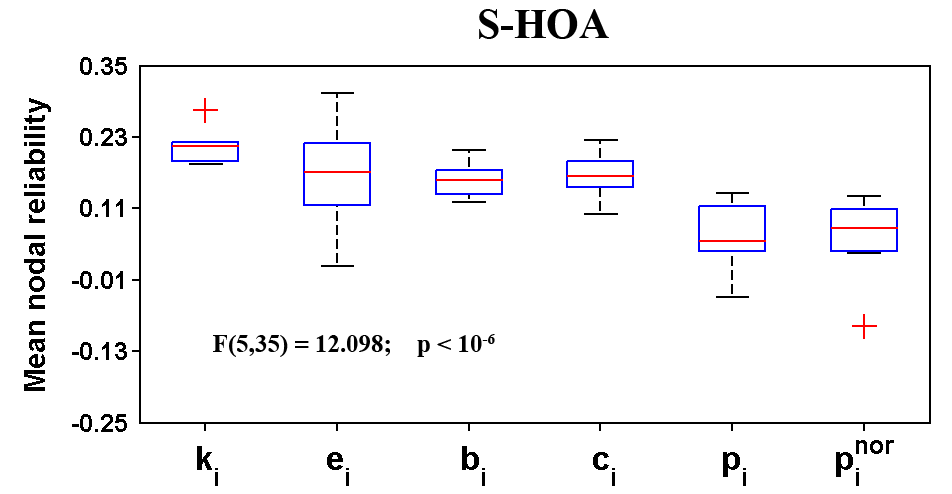


**Figure S9.** Boxplot of mean nodal TRT reliability for S-HOA-based networks


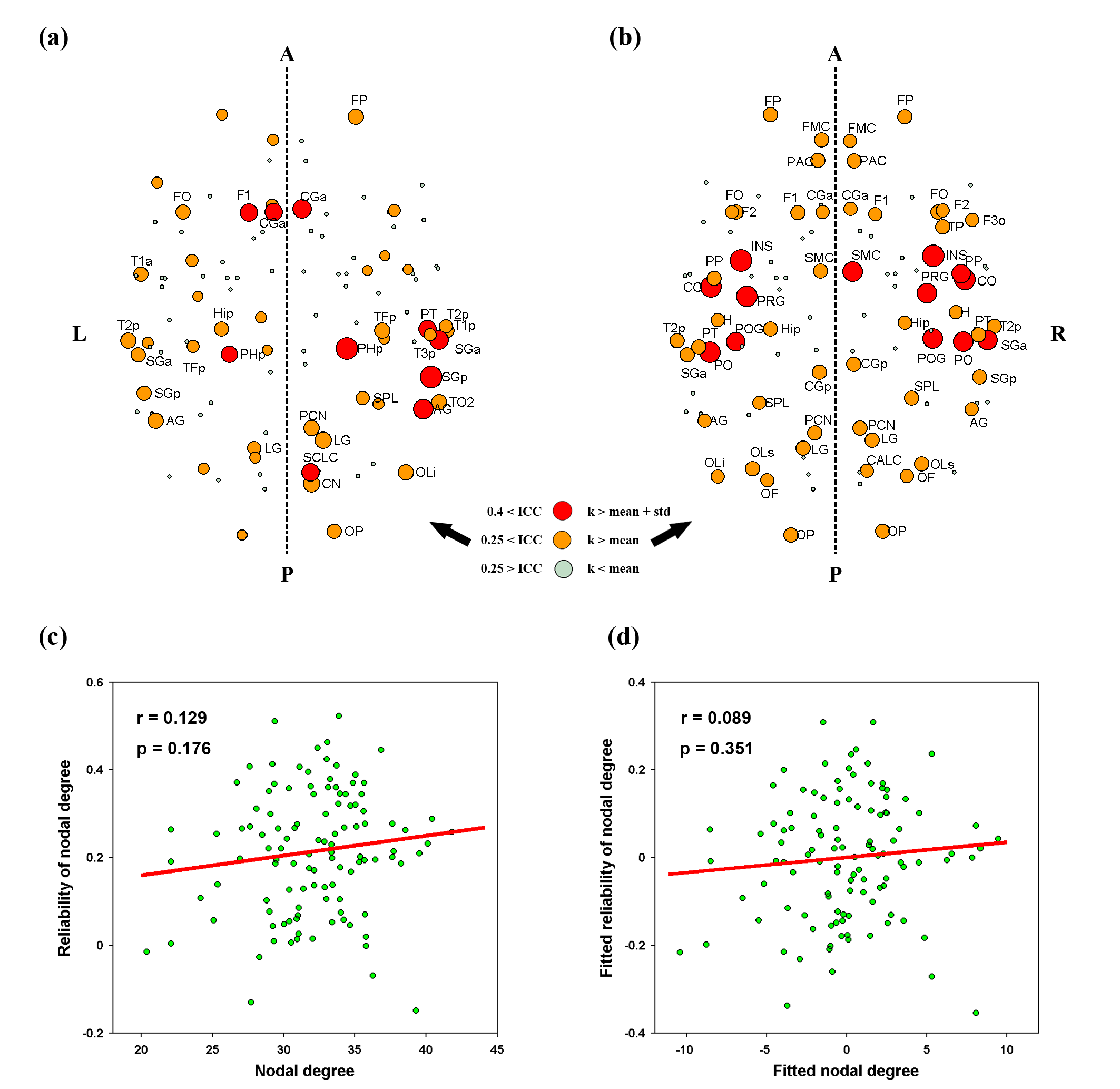


**Figure S10.** Nodal TRT reliability of degree and its relationship with nodal degree centrality for S-HOA-based networks
