# Supplementary material for: Graph Theoretical Analysis of Functional Brain Networks: Test-Retest Evaluation on Short- and Long-Term Resting-State Functional MRI Data
Source: PLoS One. 2011 Jul 19;6(7):e21976. doi: 10.1371/journal.pone.0021976 (PMC3139595; doi:10.1371/journal.pone.0021976)
Supplement: Table S1 — Regions of interest from S-AAL. (DOC) [file pone.0021976.s011.doc]

**Supporting Table S1.** Regions of interest from S-AAL

| **Index** | **Regions** | **Abbreviations** | **Index** | **Regions** | **Abbreviations** |
| --- | --- | --- | --- | --- | --- |
| 1,2 | Superior frontal gyrus, dorsolateral | SFGdor | 47,48 | Middle frontal gyrus, orbital part | ORBmid |
| 3,4 | Middle frontal gyrus | MFG | 49,50 | Inferior frontal gyrus, orbital part | ORBinf |
| 5,6 | Inferior frontal gyrus, opercular part | IFGoperc | 51,52 | Superior frontal gyrus, medial orbital | ORBsupmed |
| 7,8 | Inferior frontal gyrus, triangular part | IFGtriang | 53,54 | Gyrus rectus | REC |
| 9, 10 | Rolandic operculum | ROL | 55,56 | Insula | INS |
| 11,12 | Supplementary motor area | SMA | 57,58 | Anterior cingulate and paracingulate gyri | ACG |
| 13,14 | Superior frontal gyrus, medial | SFGmed | 59,60 | Median cingulate and paracingulate gyri | DCG |
| 15,16 | Cuneus | CUN | 61,62 | Posterior cingulate gyrus | PCG |
| 17,18 | Lingual gyrus | LING | 63,64 | Parahippocampal gyrus | PHG |
| 19,20 | Superior occipital gyrus | SOG | 65,66 | Temporal pole: superior temporal gyrus | TPOsup |
| 21,22 | Middle occipital gyrus | MOG | 67,68 | Temporal pole: middle temporal gyrus | TPOmid |
| 23,24 | Inferior occipital gyrus | IOG | 69,70 | Olfactory cortex | OLF |
| 25,26 | Fusiform gyrus | FFG | 71,72 | Hippocampus | HIP |
| 27,28 | Superior parietal gyrus | SPG | 73,74 | Amygdala | AMYG |
| 29,30 | Inferior parietal, but supramarginal  and angular gyri | IPL | 75,76 | Caudate nucleus | CAU |
| 31,32 | Supramarginal gyrus | SMG | 77,78 | Lenticular nucleus, putamen | PUT |
| 33,34 | Angular gyrus | ANG | 79,80 | Lenticular nucleus, pallidum | PAL |
| 35,36 | Precuneus | PCUN | 81,82 | Thalamus | THA |
| 37,38 | Paracentral lobule | PCL | 83,84 | Precental gyrus | PreCG |
| 39,40 | Superior temporal gyrus | STG | 85,86 | Calcarine fissure and surrounding cortex | CAL |
| 41,42 | Middle temporal gyrus | MTG | 87,88 | Postcentral gyrus | PoCG |
| 43,44 | Inferior temporal gyrus | ITG | 89,90 | Heschl gyrus | HES |
| 45,46 | Superior frontal gyrus, orbital part | ORBsup |  |  |  |

The regions are listed in terms of a prior template of Anatomical Automatic Labeling atlas (Tzourio-Mazoyer et al., 2002). Regions of left and right hemispheres are indexed by odd and even numbers, respectively.
