# Supplementary material for: Graph Theoretical Analysis of Functional Brain Networks: Test-Retest Evaluation on Short- and Long-Term Resting-State Functional MRI Data
Source: PLoS One. 2011 Jul 19;6(7):e21976. doi: 10.1371/journal.pone.0021976 (PMC3139595; doi:10.1371/journal.pone.0021976)
Supplement: Table S2 — Regions of interest from S-HOA. (DOC) [file pone.0021976.s012.doc]

**Supporting Table S2.** Regions of interest from S-HOA

| **Index** | **Regions** | **Abbreviations** | | **Index** | **Regions** | **Abbreviations** | |
| --- | --- | --- | --- | --- | --- | --- | --- |
| 1,2 | Frontal Pole | | FP | 57,58 | Cingulate Gyrus, anterior division | | CGa |
| 3,4 | Insular Cortex | | INS | 59,60 | Cingulate Gyrus, posterior division | | CGp |
| 5,6 | Superior Frontal Gyrus | | F1 | 61,62 | Precuneous Cortex | | PCN |
| 7,8 | Middle Frontal Gyrus | | F2 | 63,64 | Cuneal Cortex | | CN |
| 9, 10 | Inferior Frontal Gyrus, pars triangularis | | F3t | 65,66 | Frontal Orbital Cortex | | FOC |
| 11,12 | Inferior Frontal Gyrus, pars opercularis | | F3o | 67,68 | Parahippocampal Gyrus, anterior division | | PHa |
| 13,14 | Precentral Gyrus | | PRG | 69,70 | Parahippocampal Gyrus, posterior division | | PHp |
| 15,16 | Temporal Pole | | TP | 71,72 | Lingual Gyrus | | LG |
| 17,18 | Superior Temporal Gyrus, anterior division | | T1a | 73,74 | Temporal Fusiform Cortex, anterior division | | TFa |
| 19,20 | Superior Temporal Gyrus, posterior division | | T1p | 75,76 | Temporal Fusiform Cortex, posterior division | | TFp |
| 21,22 | Middle Temporal Gyrus, anterior division | | T2a | 77,78 | Temporal Occipital Fusiform Cortex | | TOF |
| 23,24 | Middle Temporal Gyrus, posterior division | | T2p | 79,80 | Occipital Fusiform Gyrus | | OF |
| 25,26 | Middle Temporal Gyrus, temporooccipital part | | TO2 | 81,82 | Frontal Operculum Cortex | | FO |
| 27,28 | Inferior Temporal Gyrus, anterior division | | T3a | 83,84 | Central Opercular Cortex | | CO |
| 29,30 | Inferior Temporal Gyrus, posterior division | | T3p | 85,86 | Parietal Operculum Cortex | | PO |
| 31,32 | Inferior Temporal Gyrus, temporooccipital part | | TO3 | 87,88 | Planum Polare | | PP |
| 33,34 | Postcentral Gyrus | | POG | 89,90 | Heschl's Gyrus (includes H1 and H2) | | H |
| 35,36 | Superior Parietal Lobule | | SPL | 91,92 | Planum Temporale | | PT |
| 37,38 | Supramarginal Gyrus, anterior division | | SGa | 93,94 | Supracalcarine Cortex | | SCLC |
| 39,40 | Supramarginal Gyrus, posterior division | | SGp | 95,96 | Occipital Pole | | OP |
| 41,42 | Angular Gyrus | | AG | 97,98 | Brain-Stem | | Bst |
| 43,44 | Lateral Occipital Cortex, superior division | | OLs | 99,100 | Thalamus | | Thal |
| 45,46 | Lateral Occipital Cortex, inferior division | | OLi | 101,102 | Caudate | | Caud |
| 47,48 | Intracalcarine Cortex | | CALC | 103,104 | Putamen | | Put |
| 49,50 | Frontal Medial Cortex | | FMC | 105,106 | Pallidum | | Pall |
| 51,52 | Juxtapositional Lobule Cortex (formerly Supplementary  Motor Cortex) | | SMC | 107,108 | Hippocampus | | Hip |
| 53,54 | Subcallosal Cortex | | SC | 109,110 | Amygdala | | Amy |
| 55,56 | Paracingulate Gyrus | | PAC | 111,112 | Accumbens | | Accbns |

The regions are listed in terms of a prior probability template of Harvard-Oxford atlas (Kennedy et al., 1998; Makris et al., 1999). In the current study, a threshold of 0.25 was used to determine brain area outline. Regions of left and right hemisphere are indexed by odd and even numbers, respectively.
