# Supplementary material for: Graph Theoretical Analysis of Functional Brain Networks: Test-Retest Evaluation on Short- and Long-Term Resting-State Functional MRI Data
Source: PLoS One. 2011 Jul 19;6(7):e21976. doi: 10.1371/journal.pone.0021976 (PMC3139595; doi:10.1371/journal.pone.0021976)
Supplement: Table S3 — Regions of interest from F-DOS. (DOC) [file pone.0021976.s013.doc]

**Supporting Table S3.** Regions of interest from F-Dos

| **Index** | **Regions** | **Hemisphere** | **Index** | **Regions** | **Hemisphere** | **Index** | **Regions** | **Hemisphere** | **Index** | **Regions** | **Hemisphere** |
| --- | --- | --- | --- | --- | --- | --- | --- | --- | --- | --- | --- |
| 1 | vmPFC | R | 41 | ACC | L | 81 | parietal | R | 121 | occipital | L |
| 2 | mPFC | Mid | 42 | dlPFC | R | 82 | temporal | R | 122 | occipital | L |
| 3 | aPFC | L | 43 | vPFC | L | 83 | parietal | L | 123 | occipital | R |
| 4 | vmPFC | R | 44 | dlPFC | L | 84 | sup temporal | R | 124 | temporal | R |
| 5 | vmPFC | L | 45 | dFC | R | 85 | angular gyrus | L | 125 | occipital | L |
| 6 | vmPFC | L | 46 | dFC | R | 86 | temporal | L | 126 | occipital | R |
| 7 | vmPFC | R | 47 | dFC | L | 87 | TPJ | L | 127 | occipital | R |
| 8 | ACC | R | 48 | IPL | L | 88 | frontal | R | 128 | occipital | R |
| 9 | vlPFC | R | 49 | IPL | R | 89 | dFC | R | 129 | occipital | R |
| 10 | sup frontal | R | 50 | post parietal | L | 90 | vFC | L | 130 | occipital | L |
| 11 | sup frontal | L | 51 | IPL | L | 91 | pre-SMA | R | 131 | occipital | L |
| 12 | inf temporal | R | 52 | IPL | L | 92 | vFC | R | 132 | occipital | R |
| 13 | inf temporal | L | 53 | IPL | R | 93 | SMA | Mid | 133 | occipital | R |
| 14 | post cingulate | R | 54 | IPS | L | 94 | frontal | R | 134 | occipital | R |
| 15 | fusiform | R | 55 | IPS | R | 95 | precentral gyrus | R | 135 | post occipital | L |
| 16 | precuneus | L | 56 | aPFC | R | 96 | mid insula | L | 136 | post occipital | R |
| 17 | post cingulate | L | 57 | vPFC | R | 97 | precentral gyrus | L | 137 | post occipital | R |
| 18 | inf temporal | L | 58 | ACC | L | 98 | parietal | L | 138 | post occipital | L |
| 19 | occipital | L | 59 | vFC | R | 99 | precentral gyrus | R | 139 | post occipital | L |
| 20 | post cingulate | L | 60 | ant insula | R | 100 | precentral gyrus | L | 140 | post occipital | R |
| 21 | precuneus | R | 61 | dACC | R | 101 | precentral gyrus | R | 141 | post occipital | R |
| 22 | precuneus | R | 62 | ant insula | L | 102 | parietal | L | 142 | post occipital | L |
| 23 | post cingulate | L | 63 | basal ganglia | L | 103 | mid insula | R | 143 | lat cerebellum | L |
| 24 | post cingulate | R | 64 | mFC | Mid | 104 | mid insula | L | 144 | lat cerebellum | L |
| 25 | precuneus | L | 65 | vFC | L | 105 | temporal | R | 145 | inf cerebellum | L |
| 26 | post cingulate | L | 66 | basal ganglia | L | 106 | parietal | L | 146 | lat cerebellum | L |
| 27 | angular gyrus | R | 67 | basal ganglia | R | 107 | parietal | L | 147 | med cerebellum | L |
| 28 | angular gyrus | L | 68 | vFC | L | 108 | parietal | R | 148 | inf cerebellum | L |
| 29 | precuneus | R | 69 | mid insula | R | 109 | parietal | L | 149 | inf cerebellum | R |
| 30 | IPS | L | 70 | thalamus | L | 110 | precentral gyrus | L | 150 | med cerebellum | L |
| 31 | occipital | L | 71 | thalamus | L | 111 | temporal | L | 151 | lat cerebellum | R |
| 32 | occipital | R | 72 | thalamus | R | 112 | parietal | R | 152 | med cerebellum | R |
| 33 | occipital | L | 73 | mid insula | R | 113 | post insula | R | 153 | inf cerebellum | L |
| 34 | occipital | L | 74 | mid insula | L | 114 | parietal | R | 154 | med cerebellum | L |
| 35 | aPFC | R | 75 | basal ganglia | R | 115 | parietal | L | 155 | inf cerebellum | R |
| 36 | aPFC | L | 76 | post insula | L | 116 | parietal | L | 156 | med cerebellum | R |
| 37 | vent aPFC | R | 77 | temporal | R | 117 | post parietal | L | 157 | med cerebellum | R |
| 38 | vent aPFC | L | 78 | post cingulate | L | 118 | temporal | L | 158 | inf cerebellum | L |
| 39 | vlPFC | R | 79 | fusiform | R | 119 | temporal | L | 159 | inf cerebellum | L |
| 40 | dlPFC | R | 80 | precuneus | R | 120 | sup parietal | R | 160 | inf cerebellum | R |

The regions are obtained by creating spheres (radius = 5 mm) around the peak coordinates previously identified form meta-analytic studies (Dosenbach et al., 2006; Dosenbach et al., 2010).
