# Supplementary material for: Graph Theoretical Analysis of Functional Brain Networks: Test-Retest Evaluation on Short- and Long-Term Resting-State Functional MRI Data
Source: PLoS One. 2011 Jul 19;6(7):e21976. doi: 10.1371/journal.pone.0021976 (PMC3139595; doi:10.1371/journal.pone.0021976)
Supplement: Table S4 — Correlation coefficients between long-term reliability estimated by scan1 and the average of scan 2 and scan 3 and those estimated by scan 1 and scan 3 alone. (DOC) [file pone.0021976.s014.doc]

**Supporting Table S4.** Correlation coefficients between long-term ICC values estimated by scan1 and the average of scan 2 and scan 3 and those estimated by scan 1 and scan 3 alone

|  |  |  |  | Nodal metrics | | | | | |
| --- | --- | --- | --- | --- | --- | --- | --- | --- | --- |
| Node definition | Network membership | Network type | Global metrics |  |  |  |  |  |  |
| S-AAL | Network (+/-) | Binarized | 0.552 | **0.731** | **0.729** | **0.712** | **0.547** | **0.410** | **0.303** |
|  | Network (+/-) | Weighted | **0.673** | **0.780** | **0.522** | **0.668** | **0.725** | **0.263** | **0.297** |
|  | Network (+) | Binarized | **0.729** | **0.743** | **0.697** | **0.577** | **0.618** | **0.622** | **0.587** |
|  | Network (+) | Weighted | 0.068 | **0.816** | **0.698** | **0.544** | **0.765** | **0.526** | **0.548** |
| S-HOA | Network (+/-) | Binarized | **0.753** | **0.709** | **0.729** | **0.661** | **0.567** | **0.457** | **0.543** |
|  | Network (+/-) | Weighted | 0.127 | **0.747** | **0.618** | **0.700** | **0.718** | 0.159 | 0.051 |
|  | Network (+) | Binarized | **0.777** | **0.761** | **0.761** | **0.529** | **0.636** | **0.623** | **0.643** |
|  | Network (+) | Weighted | 0.337 | **0.777** | **0.697** | **0.519** | **0.714** | **0.699** | **0.718** |
| F-DOS | Network (+/-) | Binarized | **0.796** | **0.769** | **0.775** | **0.794** | **0.659** | **0.609** | **0.580** |
|  | Network (+/-) | Weighted | 0.492 | **0.795** | **0.618** | **0.727** | **0.727** | **0.187** | 0.141 |
|  | Network (+) | Binarized | 0.121 | **0.729** | **0.637** | **0.642** | **0.714** | **0.658** | **0.632** |
|  | Network (+) | Weighted | 0.121 | **0.751** | **0.367** | **0.593** | **0.528** | **0.496** | **0.493** |

S-AAL, structural ROIs from Anatomical Automatic Labeling atlas; S-HOA, structural ROIs from Harvard-Oxford atlas; F-DOS, functional ROIs from ref (40); Network (+/-), networks constructed using absolute both positive and negative correlations; Network (+), networks constructed using only positive correlations; Binarized, binarized network analysis; Weighted, weighted network analysis. The correlation coefficients were computed across metrics for global network measures and across brain regions for nodal measures. Those significant correlations were highlighted by bold font.
