# Supplementary material for: Graph Theoretical Analysis of Functional Brain Networks: Test-Retest Evaluation on Short- and Long-Term Resting-State Functional MRI Data
Source: PLoS One. 2011 Jul 19;6(7):e21976. doi: 10.1371/journal.pone.0021976 (PMC3139595; doi:10.1371/journal.pone.0021976)
Supplement: Text S1 — Mathematical definitions of network metrics. (DOC) [file pone.0021976.s015.doc]

**Supporting Text S1. Mathematical definitions of network metrics**

Multiple network metrics of regional nodal characteristics and global network properties were calculated in the current study for both binarized networks $A=\left[a_{ij}\right]$and weighted networks $W=\left[w_{ij}\right]$. Given some network metrics are differently defined between binarized and weighted networks (e.g., clustering coefficient), for these metrics, we assigned them with superscripts B or W to differentiate binary and weighted networks, respectively. Of note, for weighted network analysis, the weights were normalized by the mean weight of all non-zero elements to achieve the same level of overall connectivity strength across population and over time. Unless otherwise mentioned, all formulas introduced below are based on $G$= ($N$,$K$), a graph or network $G$ with $N$ nodes and $K$ edges.

**Regional nodal metrics**

Six nodal measures were examined in the current study: degree $k_{i}$, clustering coefficient $c_{i}$, betweenness $b_{i}$, efficiency $e_{i}$, participant coefficient $p_{i}$, and normalized participant coefficient $p_{i}^{nor}$ . Formally, the degree of node $i$ is defined as:

$k^{B}_{i}=\sum_{j\in G} a_{ij}$ or $k_{i}^{W}=\sum_{j\in G} w_{ij}$ (1)

where $a_{ij}$($w_{ij}$) is the $(i,j)$th element in the binairzed (weighted) network of $A$($W$). Degree is a simple measurement of connectivity of a node with the rest of nodes in a network. The clustering coefficient of node $i$ is defined as [1,2]:

$c_{i}^{B}=\frac{2E}{k_{i}^{B}(k_{i}^{B}-1)}$ or $c_{i}^{W}=\frac{2}{k_{i}^{B}(k_{i}^{B}-1)}\sum_{j,k} (w_{ij}w_{jk}w_{ki})^{1/3}$ (2)

with $E$ denoting the number of edges among the nearest neighbors of $i$. Nodal clustering coefficient reflects the local interconnectivity or cliques among the neighbors of a given node. The nodal efficiency of node $i$ is computed as [3]:

$e_{i}=\frac{1}{N-1}\sum_{j\neq i\in G} \frac{1}{d_{ij}}$ (3)

where $d_{ij}$ is the shortest path length between node $i$ and node $j$ in $G$. The shortest path length is the minimum number of edges for a binarized or the smallest sum of distances for a weighted network among all possible paths from one node to another in $G$. Here, the distance between any pair of nodes $i$ and $j$ is defined as $\frac{1}{w_{ij}}$because high correlation coefficient can be interpreted as short distance between regions. Unless otherwise stated, the shortest path length in this study was calculated in the same manner as above. Nodal efficiency measures the ability of information propagation between a given node $i$ with the rest of nodes in a network. The betweenness of node $i$ is measured as [4]:

$b_{i}=\sum_{m\neq i\neq n\in G} \frac{\sigma_{mn}(i)}{\sigma_{mn}}$ (4)

where $\sigma_{mn}$ is the total number of shortest paths (paths with the shortest path length) from node $m$ to node $n$, and $\sigma_{mn}(i)$ is the number of shortest paths from node $m$ to node $n$ that pass through the node $i$. Betweenness of a node captures the influence of the node over information flow between all the other nodes in the network. Nodal participant coefficient is introduced after modularity (see below) since it is based on modular structure of a network.

**Global network metrics**

Twelve global network metrics were calculated in the current study: Small-world parameters (clustering coefficient $C_{p}$, characteristic path length $L_{p}$, normalized clustering coefficient $\gamma$, normalized characteristic path length $\lambda$ and small-worldness $\sigma$), network efficiency (global efficiency $E_{glob}$ and local efficiency $E_{loc}$), assortativity $r$, hierarchy $\beta$, synchronization $S$, modularity $Q$ and the number of modules $N_{M}$.

*Small-world parameters.* Small-world parameters of a network (clustering coefficient $C_{p}$ and characteristic path length $L_{p}$) were originally proposed by Watts and Strogatz [1]. Briefly, $C_{p}$of a network is the average of the clustering coefficients over all nodes and quantifies the local interconnectivity of a network. $L_{p}$of a network is the shortest path length required to link one node to another, averaged overall all pairs of nodes. $L_{p}$ is an indicator of overall routing efficiency of a network. In this study, we calculated $L_{p}$ as the "harmonic mean’’ distance between all possible pairs of regions [5] to deal with the disconnected graphs dilemma. To estimate the small-world properties, we scaled the $C_{p}$ and $L_{p}$ of the real brain networks with the mean $C_{p}^{rand}$ and $L_{p}^{rand}$ of 100 random networks (i.e., $\gamma=C_{p}/C_{p}^{rand}$ and $\lambda=L_{p}/L_{p}^{rand}$). Random networks were generated by a Markov-chain algorithm [6,7]. For a binary network, two pairs of nodes ($i_{1}$,$j_{1}$) and ($i_{2}$,$j_{2}$) that were connected directly were first selected at random, then if there were no edges between $i_{1}$ and $j_{2}$ and between $i_{2}$ and $j_{1}$, we removed the original two edges and added two new edges linking $i_{1}$ and $j_{2}$ and $i_{2}$ and $j_{1}$. This procedure was repeated 2 times of the number of edges in the binary network to assure the randomized organization of resultant networks. For a weighted network, the randomization was done in similar manner but in this case the weights were binding to edges. Typically, a small-world network should fulfill the following conditions: $\gamma$ > 1 and $\lambda$≈ 1 (1) and therefore the small-worldness scalar $\sigma=\gamma/\lambda$ will be more than 1 [8].

*Network efficiency.* Efficiency is a biologically plausible metric to describe brain networks from the perspective of information flow which can deal with either the disconnected or nonsparse graphs or both [9,10]. For a network $G$ with $N$ nodes and $K$edges, the global efficiency of $G$ can be computed as:

$E_{glob}(G)=\frac{1}{N(N-1)}\sum_{i\neq j\in G} \frac{1}{d_{ij}}$ (5)

where $d_{ij}$ is the shortest path length between node $i$ and node $j$ in $G$. The local efficiency of $G$is measured as:

$E_{loc}(G)=\frac{1}{N}\sum_{i\in G} E_{glob}(G_{i})$ (6)

where $E_{glob}(G_{i})$is the global efficiency of $G_{i}$, the subgraph composed of the neighbors of node $i$. Global and local efficiency measure the ability of information transmission of a network at the global and local level, respectively.

*Assortativity*. The degree correlation, $r$, is a measure of the correlation between the degree of a node and the mean degree of its nearest neighbors [11,12]. A positive value of $r>0$ means that highly connected nodes tend to connect with other nodes also with many connections, typical feature of assortative networks. In contrast, a negative value of $r<0$ implies that the highly connected nodes of a network prefer to connect to those peripheral nodes with few connections rather than each other, typical characteristic of disassortative networks. Formally, for a network with $K$ edges, the degree correlation can be measured as [11,12]:

$r^{B}=\frac{K^{-1}\sum_{i} j_{i}k_{i}-[K^{-1}\sum_{i} \frac{1}{2}(j_{i}+k_{i})]^{2}}{K^{-1}\sum_{i} \frac{1}{2}(j_{i}^{2}+k_{i}^{2})-[K^{-1}\sum_{i} \frac{1}{2}(j_{i}+k_{i})]^{2}}$ or

$r^{W}=\frac{H^{-1}\sum_{i} \varpi_{i}j_{i}k_{i}-[H^{-1}\sum_{i} \frac{1}{2}\varpi_{i}(j_{i}+k_{i})]^{2}}{H^{-1}\sum_{i} \frac{1}{2}\varpi_{i}(j_{i}^{2}+k_{i}^{2})-[H^{-1}\sum_{i} \frac{1}{2}\varpi_{i}(j_{i}+k_{i})]^{2}}$ (7)

where $j_{i}$ and $k_{i}$ are the degrees of the vertices at the ends of the $i$th edge, with $i$= 1, ... , $K$; and $\varpi_{i}$ and $H$are the weight of the $i$th edge and the total weight of all $K$edges, respectively.

*Hierarchy*. Hierarchical structure is a fundamental principle of many complex networks which could be quantified by the $\beta$ value, an exponent of the power-law relationship between clustering coefficient $C_{i}$ and degree $k_{i}$ of the nodes in a network: $C\approx k^{-\beta}$ [13]. Here, we evaluated the $\beta$ values by fitting a linear regression line to the plot of log($C$)versus log($k$) at a given threshold [14,15]. A large positive value of $\beta$ means a hierarchical network where nodes with high degree are connected predominantly to nodes not otherwise connected to each other and vice versa.

*Synchronization*. Synchronization refers to the property of a network to synchronize in dynamics among coupled oscillators. This property can be measured as

$S=\frac{\lambda_{2}}{\lambda_{N}}$ (8)

where $\lambda_{2}$ and $\lambda{}_{N}$ are the second smallest and the largest eigenvalue of the coupling matrix $G$ which is defined as [16,17]:

$G_{ij}^{B}=\delta_{ij}\sum_{i=1}^{N} a_{ij}-a_{ij}$ or $G_{ij}^{W}=\delta_{ij}\sum_{j=1}^{N} w_{ij}-w_{ij}$ (9)

*Modularity*. The modularity index $Q$ for a given partition $p$ of a brain functional network is defined as [18]:

$Q^{B}(p)=\sum_{s=1}^{N_{M}} \left[\frac{l_{s}}{L}-\left(\frac{d_{s}}{2L}\right)^{2}\right]$ or $Q^{W}(p)=\sum_{s=1}^{N_{M}} \left[\frac{w_{s}}{W}-\left(\frac{W_{s}}{2W}\right)^{2}\right]$ (10)

where $N_{M}$ is the number of modules, $L$ ($W$) is the total number of connections (total weight) in the network, $l_{s}$ ($w_{s}$) is the number of connections (the sum of the connectional weights) between nodes in module $s$, and $d_{s}$ ($W_{s}$) is the sum of $k_{i}^{B}$ ($k_{i}^{W}$) of the nodes in module $s$. Modularity quantifies the difference between the number (weight) of intra-module links of actual network and that of random network in which connections are linked (weighted) at random. The aim of the module identification process is to find a specific partition $p$ which yields the largest network modularity, $Q_{\max}$. Here, we implemented a novel spectral optimization method [19] to identify the modules that can deal with both binary and weighted networks straightforwardly using BrainConnectivityToolbox (http://www.indiana.edu/~cortex/connectivity.html). Based on the identified modular structure, the participant coefficient $p_{i}$ of a node $i$ is defined as [20,21]

$P_{i}=1-\sum_{s=1}^{N_{M}} \left(\frac{k_{is}}{k_{i}}\right)^{2}$ (11)

where $N_{M}$ is the number of modules, $k_{is}$ is the degree of node $i$ to nodes in module $s$ and $k_{i}$ is the total degree of node $i$. $P_{i}$ tends to 1 if node $i$ has a homogeneous degree distribution with all the modules and 0 if it doesn’t have any inter-module connections. Participant coefficient measures the ability of a node in keeping the communication between its own module and the other modules. Of note, the upper limit of participant coefficient for a given node is restricted by the number of identified modules, for example, for a network with four modules, the maximum of participant coefficient is $1-\sum_{s=1}^{4} \left(\frac{1}{4}\right)^{2}$= 0.75. To compensate for this effect, in the current study, we also computed the normalized participant coefficient, $p_{i}^{nor}$ for each node by dividing its possible maximum value.

Of note, most network metrics can be categorized into first-order and second-order measures according to whether or not they are dependent on more than one property or defined as rations of one property (first-order network metrics: nodal degree, , nodal clustering coefficient, , nodal efficiency, , characteristic path length, , clustering coefficient, , local efficiency, $E_{loc}$, global efficiency, $E_{glob}$ and synchronization, $S$; second-order network metrics: nodal betweenness, , nodal participant coefficient, , normalized nodal participant coefficient, , gamma, $\gamma$, lambda, $\lambda$, sigma, $\sigma$, assortativity, $r$, hierarchy, $\beta$ and modularity, $Q$).

References:

1. Watts DJ, Strogatz SH (1998) Collective dynamics of 'small-world' networks. Nature 393: 440-442.
2. Onnela JP, Saramaki J, Kertesz J, Kaski K (2005) Intensity and coherence of motifs in weighted complex networks. Phys Rev E Stat Nonlin Soft Matter Phys 71: 065103.
3. Achard S, Bullmore E (2007) Efficiency and cost of economical brain functional networks. PLoS Comput Biol 3: e17.
4. Freeman LC (1977) A Set of Measures of Centrality Based on Betweenness. Sociometry 40: 35-41.
5. Newman MEJ (2003) The structure and function of complex networks. SIAM Review 45: 167–256.
6. Maslov S, Sneppen K (2002) Specificity and stability in topology of protein networks. Science 296: 910-913.
7. Sporns O, Zwi JD (2004) The small world of the cerebral cortex. Neuroinformatics 2: 145-162.
8. Humphries MD, Gurney K, Prescott TJ (2006) The brainstem reticular formation is a small world, not scale-free, network. Proc R Soc Lond B Biol Sci 273: 503--511.
9. Latora V, Marchiori M (2001) Efficient behavior of small-world networks. Phys Rev Lett 87: 198701.
10. Latora V, Marchiori M (2003) Economic small-world behavior in weighted networks. The European Physical Journal B - Condensed Matter and Complex Systems V32: 249-263.
11. Newman ME (2002) Assortative mixing in networks. Phys Rev Lett 89: 208701.
12. Leung CC, Chau HF (2007) Weighted assortative and disassortative networks model. Physica A: Statistical Mechanics and its Applications 378: 591-602.
13. Ravasz E, Barabasi AL (2003) Hierarchical organization in complex networks. Phys Rev E Stat Nonlin Soft Matter Phys 67: 026112.
14. Supekar K, Musen M, Menon V (2009) Development of large-scale functional brain networks in children. PLoS Biol 7: e1000157.
15. Bassett DS, Bullmore E, Verchinski BA, Mattay VS, Weinberger DR, et al. (2008) Hierarchical organization of human cortical networks in health and schizophrenia. J Neurosci 28: 9239-9248.
16. Motter AE, Zhou CS, Kurths J (2005) Enhancing complex-network synchronization Europhys Lett 69: 334-340.
17. Barahona M, Pecora LM (2002) Synchronization in small-world systems. Phys Rev Lett 89: 054101.
18. Newman ME, Girvan M (2004) Finding and evaluating community structure in networks. Phys Rev E Stat Nonlin Soft Matter Phys 69: 026113.
19. Newman MEJ (2006) Finding community structure in networks using the eigenvectors of matrices. Physical Review E 74: 036104.
20. Guimera R, Nunes Amaral LA (2005) Functional cartography of complex metabolic networks. Nature 433: 895-900.
21. Guimera R, Sales-Pardo M, Amaral LAN (2007) Classes of complex networks defined by role-to-role connectivity profiles. Nat Phys 3: 63-69.
